# Reverse‐Engineered Lipid Nanoparticles Harness Protein Corona Formation for Cytokine Sequestration and Combination Immunomodulatory Therapy

**DOI:** 10.1002/smll.74914

**Published:** 2026-07-29

**Authors:** Junsik J. Sung, Gargi Yogesh Digholkar, Ryan M. Pearson

**Affiliations:** ^1^ Department of Pharmaceutical Sciences University of Maryland School of Pharmacy Baltimore Maryland USA; ^2^ Department of Microbiology and Immunology University of Maryland School of Medicine Baltimore Maryland USA; ^3^ Marlene and Stewart Greenebaum Comprehensive Cancer Center University of Maryland School of Medicine Baltimore Maryland USA

**Keywords:** anti‐inflammatory, cytokine sequestration, drug delivery, reverse‐engineered lipid nanoparticles, severe systemic inflammation

## Abstract

Cytokine storm drives the organ damage and mortality observed in sepsis, severe infection, and systemic inflammatory diseases. Despite decades of clinical effort, eliminating pathological cytokines from circulation remains an unmet medical need due to the risk of broad immunosuppression, significant toxicity, and off‐target effects. Herein, guided by lipidomic insights from cytokine‐sequestering lipid nanoparticles isolated from *Lepidium meyenii* Walp., we reverse‐engineered triglyceride‐ceramide lipid nanoparticles (TCNP) as a simplified synthetic nanoplatform that harnesses multimodal protein corona formation to broadly sequester pathological cytokines involved in systemic inflammatory disease. Rather than targeting a single cytokine axis, TCNPs were tuned to adsorb and neutralize multiple pro‐inflammatory mediators through multimodal protein corona formation. TCNP were further engineered to encapsulate dexamethasone (Dex), conferring an additional layer of intracellular immunomodulation to complement extracellular cytokine scavenging. This dual function nanoplatform exhibited selective reduction of pro‐inflammatory cytokines, including IL‐6 and TNF‐α while increasing regulatory IL‐10 and suppressing NF‐κΒ activation. In vivo, Dex‐loaded TCNP attenuated systemic inflammation, preserved organ integrity, and significantly improved survival in a lethal lipopolysaccharide‐induced endotoxemia rescue model. Collectively, these findings establish TCNP as a bioinspired nanotherapeutic platform that integrates extracellular cytokine sequestration with intracellular drug‐mediated immunomodulation to overcome key limitations of conventional anti‐inflammatory therapies.

## Graphical abstract

Bioinspired triglyceride‐ceramide lipid nanoparticles were reverse‐engineered from cytokine‐sequestering plant‐derived nanoparticles to capture pathological inflammatory mediators through protein corona formation. Dexamethasone‐loaded TCNPs combine extracellular cytokine sequestration with intracellular immunomodulation, reducing IL‐6, TNF‐α, and NF‐κΒ activation while increasing IL‐10, preserving organ integrity, and improving survival in lethal endotoxemia.

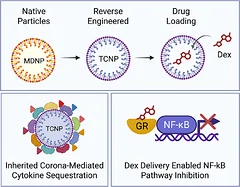

## 1 Introduction

Inflammatory diseases remain a leading cause of global morbidity and mortality and encompass diverse clinical conditions including sepsis, trauma, severe infections, and immunotherapy‐associated toxicities such as chimeric antigen receptor (CAR)‐T cell‐induced cytokine release syndrome. These disorders are driven by dysregulated host immune responses that culminate in immune dysfunction and multi‐organ failure [1, 2, 3]. Despite major advances in understanding inflammatory immunopathology, current therapeutic approaches largely focus on symptom management, and interventions directly targeting inflammatory pathways have yielded limited clinical success. Broad anti‐inflammatory strategies, including corticosteroids such as dexamethasone (Dex) [4] and methylprednisolone [5] as well as cytokine‐targeted biologics (e.g. anti‐interleukin‐6 (IL‐6) [6], anti‐tumor necrosis factor alpha (TNF‐α) [7], and anti‐IL12/23 [8]), are widely used to attenuate hyperinflammation. However, their clinical benefit remains inconsistent due to systemic toxicity, non‐specific immunosuppression, increased infection risk, and variable efficacy [9, 10]. Although these approaches can transiently reduce circulating inflammatory mediators, they often fail to produce sustained clinical benefit, highlighting fundamental limitations of therapies that target inflammation as a single downstream process rather than addressing the complex, dysregulated immune networks that drive disease progression [11, 12, 13]. These challenges highlight a critical need for therapeutic approaches capable of simultaneously modulating extracellular cytokine burden and intracellular inflammatory signaling pathways with improved specificity and cellular targeting [14].

Among inflammatory mediators, IL‐6 plays a central pathogenic role, as elevated and sustained IL‐6 signaling strongly correlates with disease severity, organ dysfunction, and mortality [15]. IL‐6 promotes pathological immune activation through pleiotropic effects on innate immune cells, endothelial function, and metabolic reprogramming, contributing to vascular leakage and tissue injury [16]. Conversely, insufficient induction of anti‐inflammatory mediators such as interleukin‐10 (IL‐10) impairs resolution of inflammation and promotes persistent immune dysregulation [17]. Clinical efforts to broadly suppress inflammation or directly neutralize IL‐6 have yielded inconsistent outcomes, reflecting the context‐dependent roles of IL‐6 in host defense and the inability of current approaches to restore balanced pro‐ and anti‐inflammatory signaling [18]. Thus, therapeutic strategies capable of simultaneously attenuating the pathological activity of IL‐6, while enhancing IL‐10‐mediated resolution may therefore offer a promising strategy to restore immune homeostasis in severe inflammatory disease.

Lipid nanoparticles (LNPs) have emerged as versatile nanotechnology platforms for therapeutic delivery, providing protection of bioactive payloads, improved pharmacokinetic profiles, and efficient cellular uptake [19]. In systemic inflammatory settings, where dysregulated cytokine production arises from complex intracellular signaling networks, LNPs can enable targeted intracellular engagement of anti‐inflammatory pathways that are incompletely accessible by free drug administration [20]. Compared to conventional systemic anti‐inflammatory agents, LNP‐based delivery offers improved control over drug exposure and cellular targeting, while minimizing broad immunosuppressive side effects [21]. As such, LNPs represent a promising strategy to enhance the therapeutic performance of anti‐inflammatory interventions.

In our previous work, we isolated bioactive LNPs from *Lepidium meyenii* Walp [22]., termed maca‐derived lipid nanoparticles (MDNP), that suppressed inflammation through a sequestration mechanism mediated by multimodal protein corona formation, and significantly improved outcomes in lethal mouse models of severe inflammation and polymicrobial sepsis [23]. However, native MDNP isolation was constrained by low yield and batch‐to‐batch variability, restricting scalability and translational development. To overcome these challenges, we reverse‐engineered a bio‐inspired synthetic analogue of MDNP guided by comprehensive lipidomic profiling, which identified triglycerides (TG) and ceramides (Cer) as dominant lipid constituents comprising more than 90% of the native MDNP composition. TG are widely used as structural cores in solid lipid nanoparticles and nanostructured lipid carriers, where they enhance the encapsulation of hydrophobic drugs, stability, and controlled release, while certain TG species possess intrinsic anti‐inflammatory and tissue‐reparative properties [24, 25]. Cer, a class of bioactive sphingolipids, can be incorporated into LNPs to improve particle uniformity and membrane stability, and regulation of inflammatory signaling pathways [26]. Together, these compositional insights provided a rational foundation for designing a simplified, reproducible, and scalable bio‐inspired nanoparticle platform that preserves the key inflammation scavenging immunomodulatory properties of native MDNP, while enabling tunable therapeutic drug delivery.

Here, we report the formulation, optimization, physicochemical characterization, and therapeutic development of MDNP‐inspired, reverse‐engineered triglyceride‐ceramide lipid nanoparticles (TCNP) as a dual‐function inflammation‐scavenging and enhanced drug delivery system for treatment of severe inflammatory disease (Figure 1). TCNP exhibited robust inflammatory cytokine sequestration abilities with comparable multimodal protein corona formation compared to MDNP. Subsequently, dexamethasone was encapsulated (Dex‐TCNP) to evaluate the synergistic dual function therapeutic activity using in vitro and in vivo rescue models of lipopolysaccharide (LPS)‐induced endotoxemia. Dex‐TCNP treatment of lipopolysaccharide (LPS)‐induced macrophages led to significant reductions in pro‐inflammatory mediators and nuclear factor‐kappa beta (NF‐κβ) activity compared to concentration matched TCNP or Dex only controls. Transcriptional analysis using NanoString identified *Il6* as the major inflammatory gene suppressed by TCNP, which was further enhanced by Dex‐TCNP compared to Dex. Therapeutic administration of Dex‐TCNP to LPS‐challenged mice resulted in significant reductions in circulating plasma IL‐6 and TNF‐α, with a notable increase in anti‐inflammatory IL‐10. These systemic improvements in cytokine modulation led to marked reductions in organ injury scores and significantly improved survival compared to controls. By establishing the design parameters and mechanism for an effective dual function inflammation scavenging drug delivery system, this work establishes TCNP as a translational therapeutic nanoplatform to treat a diverse set of severe inflammatory diseases.

**FIGURE 1:**
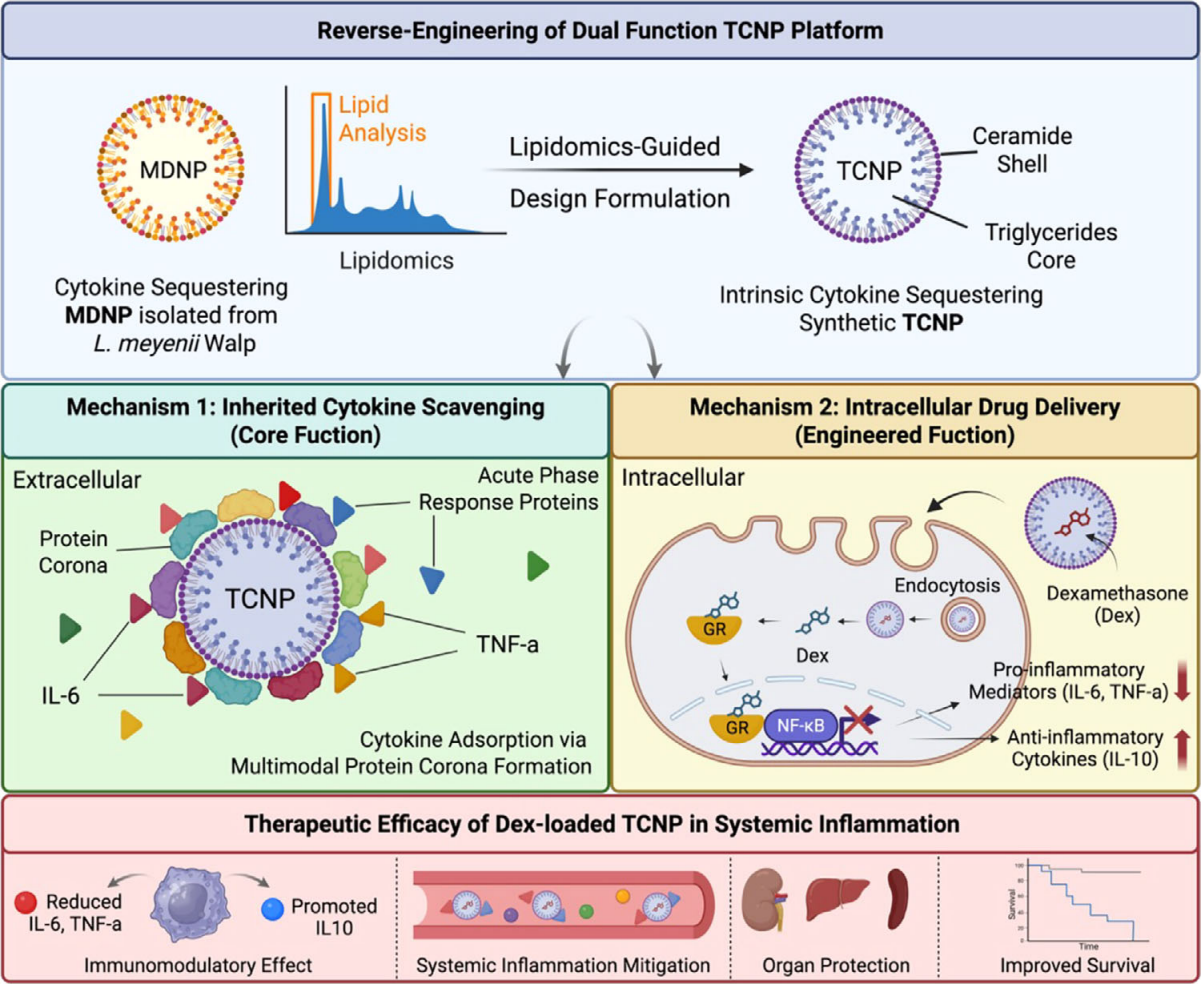
Schematic illustration depicting the reverse‐engineering of TCNP from MDNP with a dual anti‐inflammatory mechanism for treating systemic inflammation. Under systemic inflammation, dexamethasone (Dex)‐loaded TCNPs (Dex‐TCNP) simultaneously sequester pro‐inflammatory cytokines and deliver Dex intracellularly. This dual action dampens pre‐existing proinflammatory mediators in circulation, while inhibiting transcription factor activity to collectively reduce pro‐inflammatory cytokines, increase anti‐inflammatory cytokines, promote organ recovery, and improve survival. Created in BioRender. Sung, J. (2026) https://BioRender.com/t298t39.

## 2 Results

### 2.1 Preparation and Characterization of TCNP

Our previous research isolated MDNP from *L. meyenii* Walp., performed untargeted lipidomic analysis to determine their structural composition, and determined their inherent anti‐inflammatory mechanism and therapeutic efficacy for treating sepsis [23]. From the lipidomics data, TG and Cer comprised greater than 90% of the MDNP composition in an approximately 3:1 TG:Cer ratio. To develop a bio‐inspired TCNP, individual TG and Cer components were combined at defined ratios of 75 TG:25 Cer to replicate the dominant lipid composition and physicochemical features of MDNP. Transmission electron microscopy (TEM) revealed that TCNP exhibited spherical morphology with a smooth and uniform outer surface compared to freshly isolated MDNP (Figure 2A). To understand how varying TG‐to‐Cer ratios (75:25, 50:50, and 25:75) and formulation temperatures (40°C or 75°C) impacted TCNP physicochemical properties, we prepared a set of six types of TCNP using the thin film hydration method. Dynamic light scattering demonstrated that the 75:25 TG‐Cer formulation extruded at 40°C yielded particles with an average hydrodynamic diameter of 137.9 nm, a zeta potential of −14.3 mV, and a polydispersity index (PDI) of 0.213, recapitulating key physicochemical features of native MDNP (Table 1). Particles prepared with 50:50 or 25:75 TG‐Cer ratios resulted in increased sizes and polydispersity, while extrusion at 75°C led to further particle heterogeneity, likely due to thermal instability of the lipid blend (Figure 2B, Figure S1A–C). Collectively, these results identified the 75:25 TG‐Cer TCNP formulation extruded at 40°C as the most stable and reproducible composition. Lastly, we performed stability studies using TCNP over 10 days, where it was confirmed that TCNP stored at 4°C maintained its size and zeta potential with minimal aggregation, whereas room temperature storage resulted in progressive instability as demonstrated by inconsistent size distributions beyond day 3 (Figure 2C,D).

**FIGURE 2:**
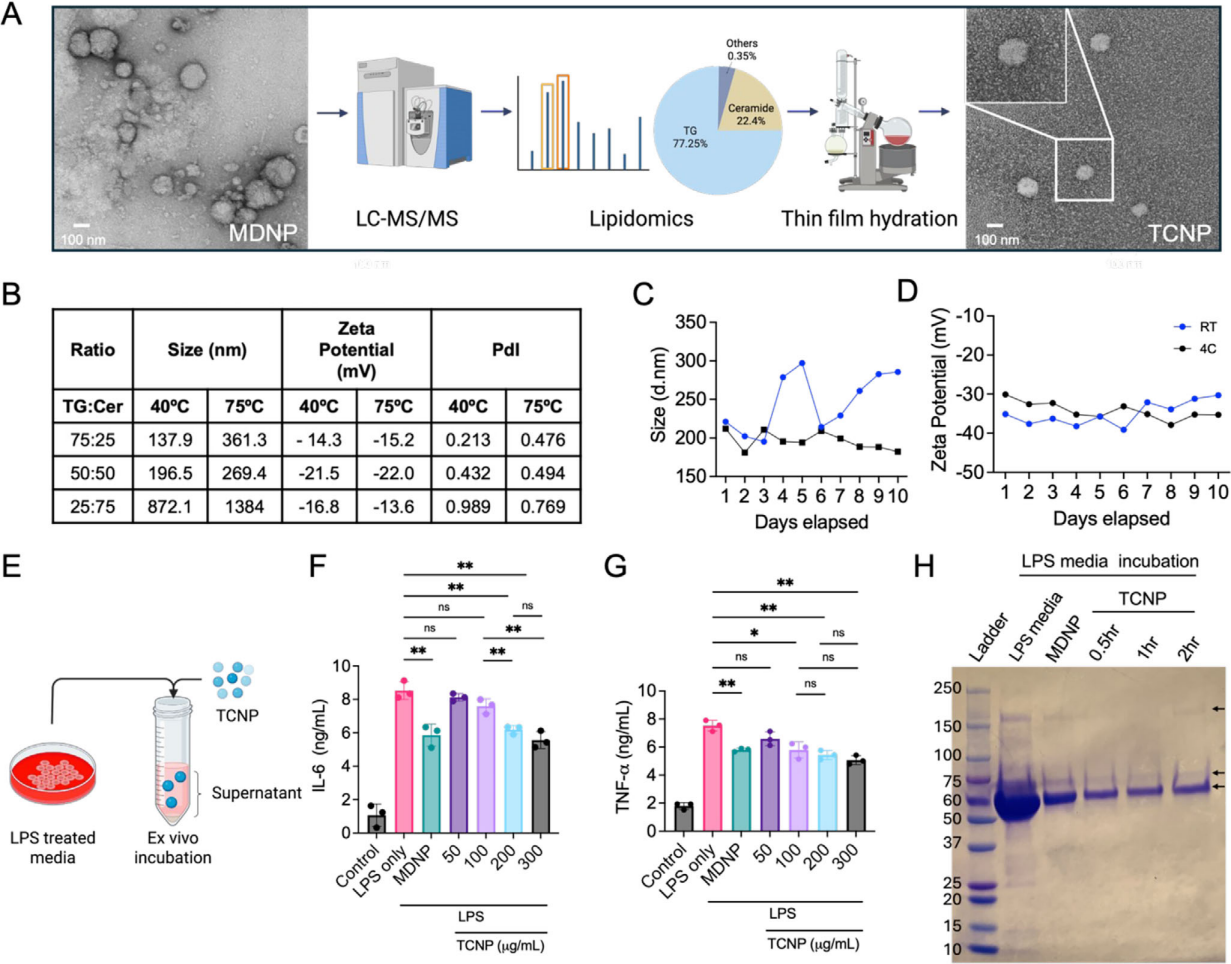
Preparation and characterization of TCNP. (A) A novel TG‐Cer LNP was synthesized based on the lipid composition of maca‐derived lipid nanoparticle (MDNP), as visualized by TEM. Scale bar = 100 nm. Created in BioRender. Sung, J. (2026) https://BioRender.com/5zij92n. (B) TG‐Cer LNP were formulated at various TG‐Cer ratios and characterized for particle size and zeta potential following extrusion at 40°C and 75°C. (C,D) Stability of optimized TCNP formulation was confirmed at both RT and 4°C up to 10 days. (E) Schematic illustration of ex vivo sequestration test of TCNP binding to LPS‐induced cytokines Reduction of pro‐inflammatory cytokines were measured in (F) IL‐6 and (G) TNF‐α. Created in BioRender. Sung, J. (2026) https://BioRender.com/obegm63. (H) SDS‐PAGE gel image confirms the formation of protein corona fingerprint similar to MDNP. *p < 0.05, **p < 0.01 versus LPS control group.

**TABLE 1:** Comparative physicochemical and functional characterization of MDNP and TCNP. Key properties of native MDNP and reverse‐engineered TCNP with and without Dex encapsulation are summarized, including particle size, zeta potential, polydispersity (PDI), lipid composition, cytokine sequestration efficiency, protein corona formation, drug loading capability and scalability.

Property	MDNP (Native)	TCNP (Reverse‐Engineered)
Particle Size (nm)	174.5 nm	137.9 nm (167.9–172.8 nm with Dex)
PDI	0.2	0.213 (0.171–0.201 with Dex)
Zeta Potential (mV)	−9.6 mV	−14.3 mV (−16.3–−12.2 mV with Dex)
Morphology (TEM)	Spherical, Heterogeneous Surface	Spherical, Smooth Uniform Surface
Lipid composition	TG, Ceramide, FFA, DGDG, LPC, PC, PI	TG, Ceramide
Stability (4°C)	Maintained up to 10 days	Maintained up to 10 days
Protein Corona Formation	Yes (Proteomics confirmed)	Yes (Conserved fingerprint from SDS‐PAGE)
Cytokine Sequestration	Yes	Yes
Drug Encapsulation	Not reported	Dexamethasone
Production Scalability	Limited	Scalable
Batch‐to‐Batch Variability	High	Low

### 2.2 In Vitro Evaluation of Pro‐Inflammatory Cytokine Sequestration Through Protein Corona Formation

The previously described primary anti‐inflammatory mechanism of MDNP was identified as the sequestration of pro‐inflammatory mediators through multimodal protein corona formation. Based on this principle, we hypothesized that TCNP would similarly adsorb pro‐inflammatory cytokines as part of its protein corona. To evaluate this, TCNP was incubated with LPS‐stimulated macrophage culture media, which contains elevated concentrations of multiple pro‐inflammatory cytokines (Figure 2E). ELISA quantification following TCNP removal via centrifugation demonstrated that TCNP incubation in inflammatory cell culture media reduced the levels of interleukin‐6 (IL‐6) and tumor necrosis factor‐alpha (TNF‐α) in a concentration‐dependent manner (Figure 2F,G). Among tested concentrations, 200 µg/mL TCNP showed the most effective and efficient suppression of IL‐6 and TNF‐α, while increasing the concentration to 300 µg/mL did not significantly enhance the anti‐inflammatory effects. The absence of additional significant cytokine reductions at higher concentrations suggests a plateau in the dominant saturable cytokine sequestration mechanism, potentially compounded by concentration‐dependent changes in particle‐protein interaction that reduce effective bioavailable surface area. Therefore, 200 µg/mL was selected as the optimal concentration for subsequent in vitro studies. Although the reduction was significant, the overall sequestration efficiency was less than that observed for MDNP (100 µg/mL), suggesting that differences in lipid composition and surface properties influenced the affinity of TCNP for cytokine binding.

We next evaluated the protein corona formed on TCNP in comparison to MDNP using SDS‐PAGE. TCNP and MDNP were incubated in LPS‐conditioned media for up to 2 h prior to corona isolation. Dynamic light scattering revealed an increase in diameter following incubation in LPS‐conditioned media, confirming the formation of a protein corona (Figure S2). A similar protein fingerprint was observed for all samples, suggesting a conserved corona‐forming capacity across both platforms (Figure 2H). To further substantiate that the observed cytokine depletion reflected physical sequestration by TCNP rather than assay interference, degradation, or non‐specific loss, cytokines were quantified in the corona‐dissociated TCNP pellet fraction following incubation with LPS‐stimulated BMDM‐conditioned media. IL‐6 and TNF‐α were both detectable in the TCNP‐associated pellet fraction, demonstrating direct recovery of cytokines from the TCNP corona. When normalized to the amount of cytokine depleted from the corresponding supernatant, 47.6% of depleted IL‐6 and 41.5% of depleted TNF‐α were recovered from the TCNP pellet fraction. These pellet‐recovery data provide orthogonal evidence that TCNP physically associates with inflammatory cytokines and supports TCNP‐mediated cytokine sequestration as a contributor to the reduced soluble cytokine levels observed in conditioned media (Figure S3). Taken together, these findings demonstrate that TCNP was successfully engineered to sequester pro‐inflammatory cytokines, with consistent protein corona formation as MDNP, confirming the potential for TCNP to serve as a suitable and bio‐inspired nanotherapy to mitigate severe inflammation.

### 2.3 Engineering TCNP for Enhanced Immunomodulation

We next assessed if TCNP could be engineered to serve as an enhanced immunomodulatory delivery platform capable of simultaneously sequestering pro‐inflammatory cytokines, while encapsulating Dex, a widely used glucocorticoid with potent anti‐inflammatory activity (Figure 3A). Dex was selected given its established ability to suppress IL‐6 signaling through NF‐κβ inhibition, enabling investigation of potential synergy between intracellular glucocorticoid‐mediated inhibition and TCNP‐mediated extracellular cytokine modulation [27]. Specifically, TCNP loaded with 0.8 ng/mg Dex (Dex‐TCNP^L^) exhibited an average size of 167.9 nm, zeta potential of −12.2 mV, and PDI of 0.171; particles loaded with 8 ng/mg (w/w) Dex (Dex‐TCNP^M^) measured 171.8 nm, −15.6 mV, and PDI of 0.213; and those loaded with 80 ng/mg (w/w) Dex (Dex‐TCNP^H^) measured 172.8 nm, −16.3 mV, and PDI of 0.201 (Figure 3B). The encapsulation efficiency of three different loadings (Dex‐TCNP^L^, ‐TCNP^M^, ‐TCNP^H^) was comparable, with average values of 54.1%, 53.2%, and 54.4%, respectively (Figure 3C), and physicochemical characterization showed stable particle size and surface charge across multiple drug‐loadings. These results demonstrate consistent stability and low polydispersity across all tested formulations.

**FIGURE 3:**
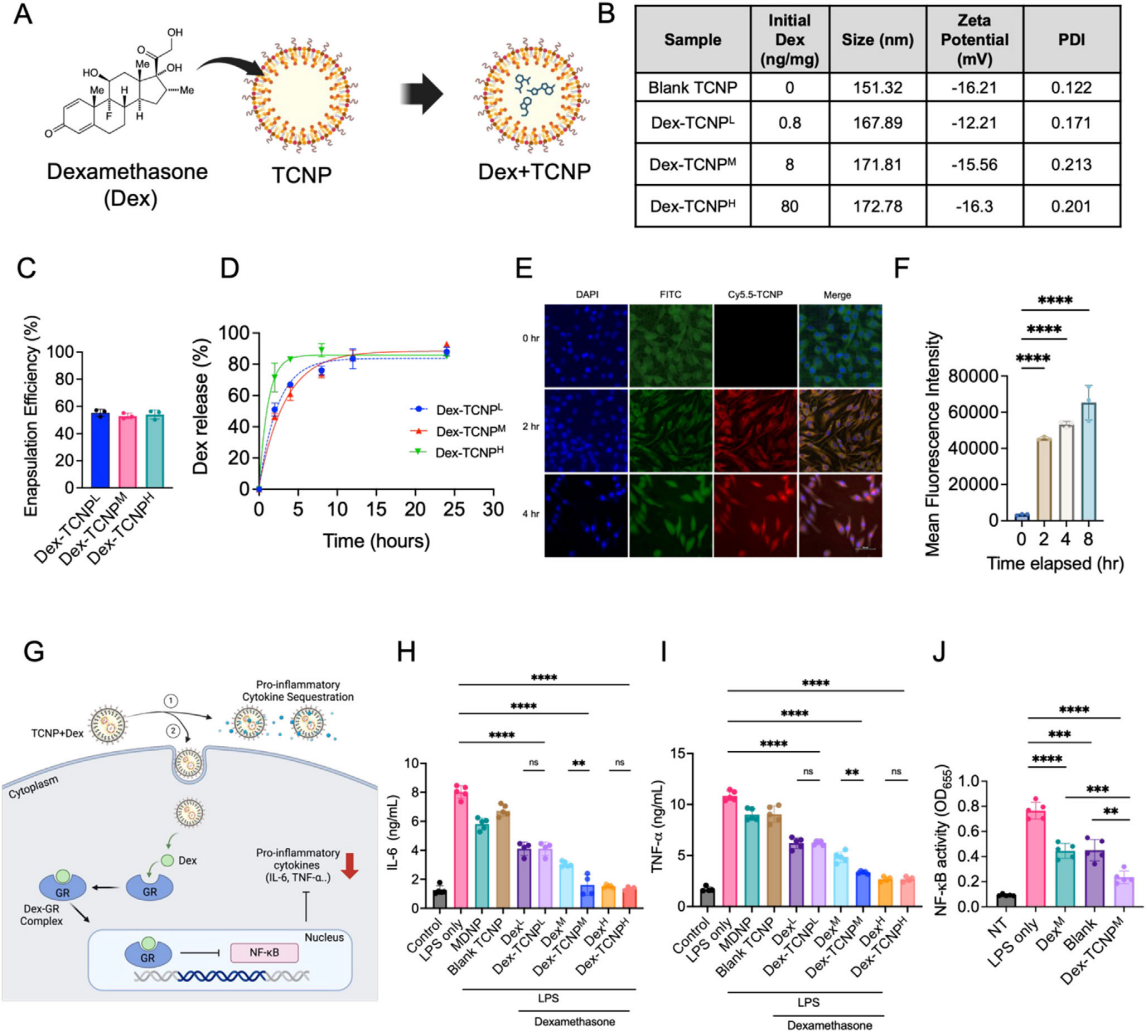
Characterization of Dex‐encapsulating TCNP. (A) A graphical summary illustrating the formulation of the Dex‐TCNP drug delivery system. Created in BioRender. Sung, J. (2026) https://BioRender.com/lw593fe. (B) Evaluation of size, zeta potential, PDI of various formulations of Dex‐TCNP prepared by encapsulating increased amounts of Dex. (C) Encapsulation efficiency of Dex within Dex‐TCNP. (D) Release profile of Dex from Dex‐TCNP in PBS over 24 h. (E) Time‐dependent uptake of Cy5.5 labeled‐Dex‐TCNP by BMDM. (F) Quantitative mean fluorescence intensity measurement of cellular uptake via flow cytometry. (G) Dual in vitro mechanism for reducing pro‐inflammatory cytokines through sequestration and Dex‐GR complex‐mediated inhibition of NF‐κΒ pathway. Created in BioRender. Sung, J. (2026) https://BioRender.com/zoaq9jj. BMDM were treated with LPS (100 ng/mL) for 4 h prior to treatment with various groups for 8 h. ELISA was used to assess levels of (H) IL‐6 and (I) TNF‐α. (J) NF‐κΒ activity was determined by treating LPS‐activated (4 h) RAW‐Blue cells for 8 h with various treatments. *p < 0.05, **p < 0.01, ***p < 0.001, and ****p < 0.0001 versus LPS control group.

### 2.4 Functional Evaluation of Dexamethasone‐Loaded TCNP

Drug release studies further indicated a sustained and controlled release profile of Dex from Dex‐TCNP across all variants tested, supporting their potential for prolonged anti‐inflammatory activity (Figure 3D). Cellular uptake studies using Cyanine 5.5 (Cy5.5) fluorescently labeled TCNP revealed a time‐dependent increase in internalization by bone marrow‐derived macrophages (BMDM) (Figure 3E,F). Cytotoxicity assessment using lactate dehydrogenase (LDH) release assays showed minimal toxicity (Figure S4). Given that TCNP can both interact with inflammatory mediators in the extracellular milieu and serve as an efficient drug carrier, we next investigated whether Dex‐TCNP exerted an enhanced and dual anti‐inflammatory activity through extracellular cytokine sequestration and intracellular drug delivery (Figure 3G). Blank TCNP alone reduced IL‐6 and TNF‐α levels in LPS‐stimulated BMDM, confirming that the TCNP platform retains inherited cytokine‐suppressing activity in a cellular context (Figure 3H,I). Dex‐loading further enhanced this suppression, with Dex‐TCNP^M^, achieving significantly improved anti‐inflammatory effects compared to both blank TCNP and concentration‐matched free Dex. Blank TCNP also modestly suppressed NF‐κΒ activation, while Dex‐TCNP^M^ produced a significantly greater reduction consistent with additive anti‐inflammatory activity (Figure 3J). Although the higher Dex‐loaded formulation (Dex‐TCNP^H^) produced comparable reductions for pro‐inflammatory cytokines, no additional benefit was observed relative to free Dex^H^ or Dex‐TCNP^M^. Given the equivalent efficacy, the Dex‐TCNP^M^ formulation was selected to minimize unnecessary nanoparticle exposure and reduce the potential for off target or unintended effects. These findings suggest that TCNP not only provides an efficient nanoplatform for drug encapsulation and delivery but also possess inherited anti‐inflammatory activity that is meaningfully amplified by Dex encapsulation, combining direct drug activity with TCNP‐mediated sequestration of inflammatory mediators.

### 2.5 Comprehensive NanoString‐Based Transcriptional Analysis

To comprehensively evaluate transcriptional reprogramming induced by TCNP and Dex‐TCNP, gene expression profiling was performed using NanoString analysis of a 254‐gene inflammation panel in LPS‐challenged bone marrow–derived macrophages (BMDM). Total RNA was isolated following treatment, and differential gene expression was analyzed relative to LPS‐treated controls (Figure 4A). Heatmap visualization revealed distinct transcriptional signatures across treatment groups. Both free Dex and Dex‐TCNP produced substantial downregulation of LPS‐induced inflammatory gene expression, with the most pronounced suppression observed in Dex‐containing formulations. Importantly, blank TCNP also generated a distinct transcriptional profile compared to LPS‐treated cells, demonstrating intrinsic immunomodulatory activity independent of drug loading (Figure 4B).

**FIGURE 4:**
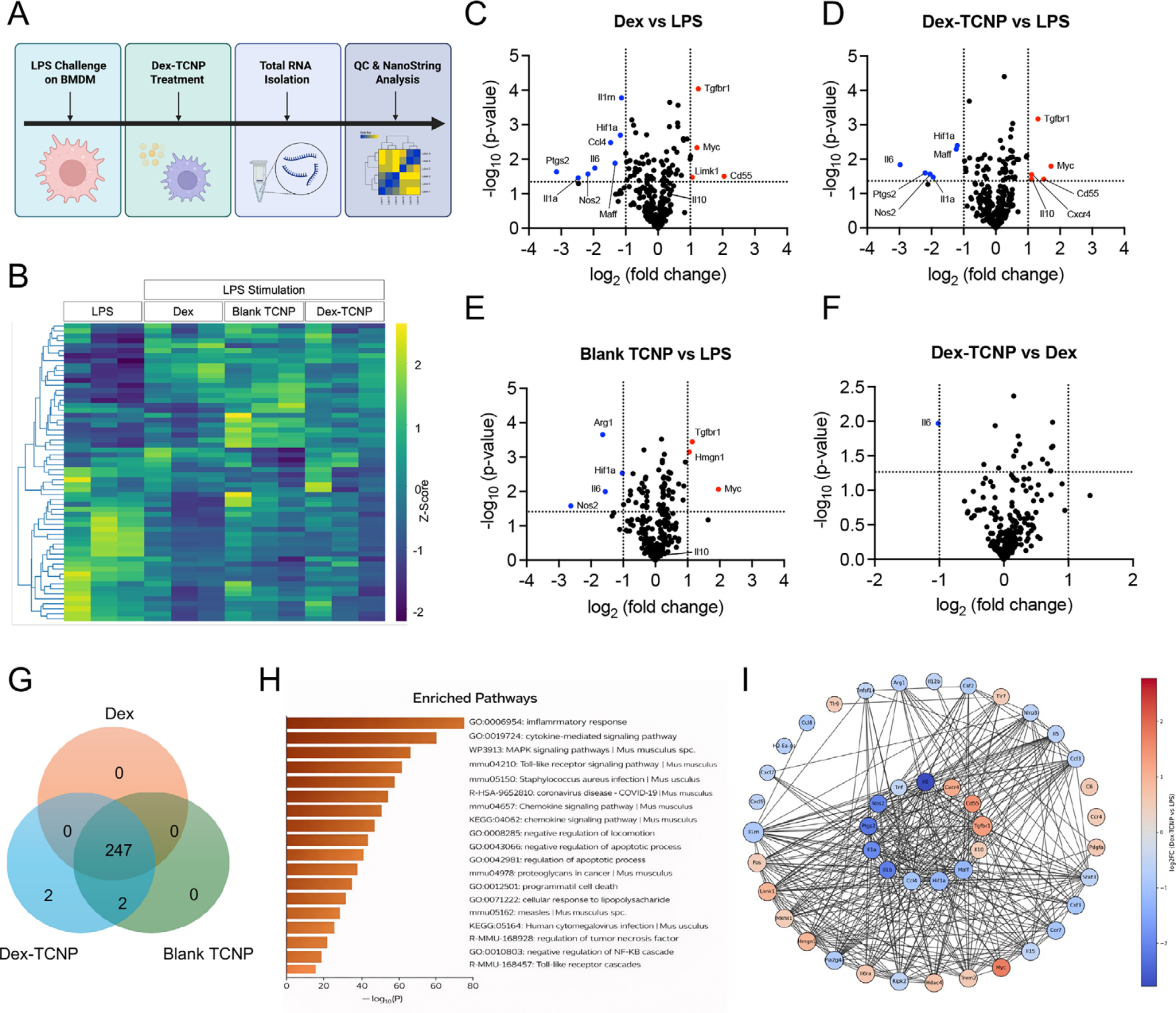
Transcriptional analysis of Dex‐TCNP under severe inflammation. LPS‐activated BMDMs (4 hrs) were treated with Dex, Blank TCNP, and Dex‐TCNP for 8 hrs prior to NanoString analysis of 254 inflammatory genes. (A) Workflow of the NanoString analysis using purified total RNA from LPS induced BMDM after NP treatments. Created in BioRender. Sung, J. (2026) https://BioRender.com/v2k4qgd. (B) Heatmap representation displaying hierarchical clustering of various inflammatory genes expressed in response to various treatments. (C‐F) Volcano plots of linear counts comparing against LPS treated group and Dex vs Dex‐TCNP. (G) Venn diagram displaying 247 genes are common among all groups and 2 distinct genes (*Creb1* and *Areg*) were expressed in Dex‐TCNP group. (H) Top 20 pathway and process enrichment analysis using nSolver and Metascape. (I) Gene network coordinating transcriptional reprogramming induced by Dex‐TCNP (*n* = 3 for each group).

Volcano plots were prepared for each treatment group relative to LPS controls. Free Dex treatment significantly downregulated canonical inflammatory genes including *Il1a*, *Il6*, *Hif1a*, *Ccl4*, *Nos2*, and *Ptgs2*, while upregulating regulatory genes such as *Tgfbr1*, *Myc*, *Limk1*, and *Cd55* (Figure 4C). Dex‐TCNP produced a similar but enhanced anti‐inflammatory transcriptional profile, characterized by strong suppression of *Hif1a*, *Maff*, *Il6*, *Il1a*, and *Ptgs2*, along with increased expression of regulatory genes including *Tgfbr1*, *Myc*, *Il10*, and *Cd55* (Figure 4D). Notably, blank TCNP alone elicited a distinct transcriptional response marked by upregulation of *Tgfbr1*, *Hmgn1*, and *Myc*, together with downregulation of *Arg1*, *Hif1a*, *Il6*, and *Nos2*, indicating carrier‐driven immunomodulation independent of Dex (Figure 4E). Although Dex‐TCNP did not produce widespread additional transcriptional suppression relative to free Dex, it selectively enhanced downregulation of *Il6* and upregulation of *Il10*. This focused transcriptional shift suggests that Dex‐TCNP does not simply amplify global anti‐inflammatory signaling but instead rebalances inflammatory responses by simultaneously suppressing a key pathogenic cytokine, while promoting pro‐resolving regulatory pathways.

Given the well‐established role of sustained IL‐6 signaling and driving inflammatory disease severity, organ dysfunction, and mortality, the pronounced reduction of *Il6* combined with increased *Il10* expression following Dex‐TCNP treatment, represents a therapeutically meaningful enhancement for managing inflammation. Interestingly, selective downregulation of *Arg1* and upregulation of *Hmgn1* were uniquely observed in the blank TCNP group, defining a distinct platform‐specific transcriptional signature. Because Arg1 [28] and Hmgn1 [29] are associated with macrophage functional polarization and chromatin‐mediated transcriptional regulation, respectively, these findings suggest that TCNP engages immune regulatory programs that are mechanistically distinct from glucocorticoid signaling. Taken together, these results indicate that blank TCNP engages an immunomodulatory transcriptional program independent of drug cargo, and that Dex loading builds upon this foundation to produce a qualitatively distinct and therapeutically enhanced response characterized by focused *Il6* suppression and *Il10* induction. Furthermore, the enhanced anti‐inflammatory effects observed with Dex‐TCNP are consistent with improved intracellular delivery and pharmacological activity enabled by TCNP‐mediated drug delivery.

Venn diagram analysis demonstrated that 247 genes were commonly regulated across treatment groups, whereas Dex‐TCNP uniquely regulated 2 genes including *Creb1* and *Areg* (Figure 4G). *Creb1* encodes a transcriptional factor known to suppress pro‐inflammatory signaling by antagonizing NF‐κΒ activity and promoting macrophage tolerance, while *Areg* is associated with tissue repair processes [30, 31]. Additionally, *Fasl* and *Il17a* were selectively regulated by both blank TCNP and Dex‐TCNP treatments but not free Dex, indicating TCNP‐mediated transcriptional effects. *Fasl* is involved in immune cell apoptosis and homeostatic regulation, while *Il17a* encodes a cytokine that mediates immune communication and coordination between innate and adaptive immune responses [32, 33]. The regulation of these genes in the absence of broad pro‐inflammatory gene induction suggests that TCNP‐based formulations preferentially promote immune regulatory signaling rather than classical inflammatory polarization. Together, these findings support a model in which Dex‐TCNP integrates TCNP‐mediated immunomodulation with cargo‐driven signaling to produce a distinct transcriptional response.

To further interpret the biological significance of these transcriptional changes, pathway enrichment analysis was performed using Metascape (version 3.5) [34]. Consistent with observed therapeutic effects, enriched pathways were strongly associated with inflammatory responses, cytokine signaling, and immune regulation (Figure 4H). Notably, enrichment of pathways involved in negative regulation of NF‐κΒ signaling, chemokine signaling, and apoptosis suggests that Dex‐TCNP not only suppresses pro‐inflammatory activation but also engages feedback mechanisms that limit excessive immune signaling and promote inflammatory resolution. Gene network analysis revealed an IL‐6‐centered inflammatory module that was broadly suppressed by Dex‐TCNP treatment. Downregulated genes, including *Il6*, *Tnfa*, *Nos2*, *Ptgs2*, *Il1a/b*, *Ccl4*, *Hif1a*, and *Maff*, formed a tightly connected network, indicating coordinated attenuation of canonical LPS‐driven signaling. Concurrently, upregulation of regulatory genes such as *Il10*, *Tgfbr1*, *Cxcr4*, and *Cd55* indicated a shift toward an immunoregulatory transcriptional phenotype (Figure 4I).

### 2.6 In Vivo Biodistribution and Anti‐Inflammatory Effect of Dex‐TCNP

To define the tissue targeting properties of TCNP and evaluate its therapeutic potential in systemic inflammation, biodistribution studies were performed using fluorescently labeled blank TCNP and Dex‐TCNP, followed by efficacy assessment in a murine lipopolysaccharide (LPS)‐induced endotoxemia model (Figure 5A). Two doses of each treatment were administered during the early phase following LPS challenge to maintain therapeutic exposure during the peak cytokine surge and suppress the rapidly evolving acute inflammatory response. After 4 hrs, biodistribution was evaluated by IVIS imaging, and plasma cytokines and tissue histology were analyzed. Both formulations preferentially accumulated in the liver and kidney following intraperitoneal administration, with minimal accumulation in other organs including the lung, spleen, and heart, independent of Dex loading, consistent with known clearance and uptake pathways of MDNP (Figure 5B,C) [23]. Systemic cytokine levels were quantified using a multiplex Luminex assay. Dex‐TCNP markedly suppressed key pro‐inflammatory cytokines, including IL‐6 and TNF‐α, confirming potent anti‐inflammatory activity. Blank TCNP significantly reduced IL‐6 but did not significantly alter TNF‐α or IL‐10 levels, indicating preferential cytokine modulation by the TCNP platform itself. Free Dex suppressed both IL‐6 and TNF‐α but did not significantly elevate IL‐10. In contrast, Dex‐TCNP significantly increased IL‐10 levels, consistent with NanoString findings (Figure 5D–G). Blood biochemistry analysis further demonstrated that Dex‐TCNP reduced ALT and BUN levels, indicating attenuated hepatic and renal injury comparable to free Dex and superior to blank TCNP (Figure S5). Together, the coordinated suppression of pro‐inflammatory cytokines and induction of IL‐10 indicate that Dex‐TCNP promotes immune resolution rather than broad immunosuppression, thereby mitigating systemic inflammation and associated organ injury in LPS‐challenged mice.

**FIGURE 5:**
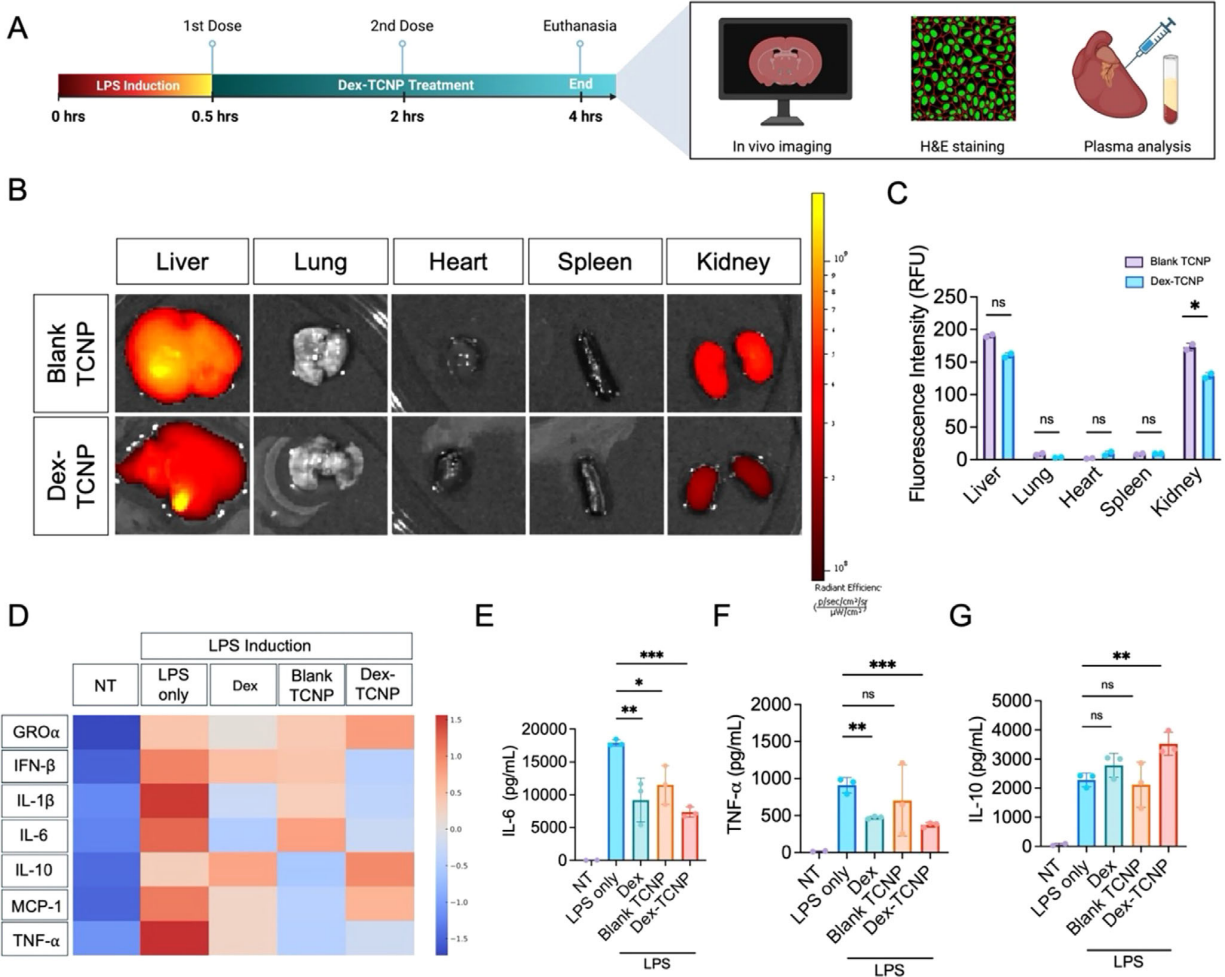
Biodistribution and anti‐inflammatory effects of Dex‐TCNP in a therapeutic LPS‐induced endotoxemia mouse model. (A) A timeline for drug delivery treatment of Dex‐TCNP after LPS challenge. Created in BioRender. Sung, J. (2026) https://BioRender.com/n92o995. (B) IVIS image demonstrating the biodistribution of intraperitoneally delivered Blank and Dex‐TCNP in liver, lung, heart, spleen and kidney. (C) Fluorescence intensity quantitation of the Cy5.5‐labeled TCNP. (D) Heatmap of cytokine expression including GROα, IFN‐β, IL‐1β, IL‐6, IL‐10, MCP‐1, and TNF‐α. A significant expression reduction was observed in (E) IL‐6 (F) TNF‐α and increase in (G) IL‐10 Data are expressed in mean ± SD (*n* = 3) *p < 0.05, **p < 0.01, ***p < 0.001 versus LPS control group.

### 2.7 In Vivo Assessment of Organ Protection and Survival

To evaluate the efficacy of Dex‐TCNP in mitigating inflammation‐induced multi‐organ damage, histological analysis was performed on major organs (liver, kidney, spleen, lung, and heart) collected following LPS challenge and treatment. Representative H&E‐stained sections are shown in Figure 6A, and quantitative injury scores were calculated (Figure 6B–F). As expected, LPS‐treated mice exhibited extensive pathological alterations across multiple organs, including hepatocellular necrosis and sinusoidal congestion in the liver, tubular degeneration and necrosis in the kidney, white pulp disorganization in the spleen, and severe pulmonary injury characterized by alveolar collapse and inflammatory cell infiltration. These findings corresponded to high histological injury scores across most organs. Treatment with free Dex partially attenuated these pathological changes, reducing necrosis and inflammatory infiltration, although moderate injury persisted, particularly in the spleen and lungs. In contrast, Dex‐TCNP treatment markedly preserved tissue architecture across all examined organs. Histological evaluation revealed minimal necrosis, reduced inflammatory infiltration, and largely intact organ morphology, consistent with near‐baseline injury scores. Direct comparison of blank TCNP and Dex‐TCNP injury scores confirms that drug loading substantially amplifies the tissue‐protective capacity of the TCNP platform, achieving near‐base‐line preservation across all five organs not observed with blank TCNP alone. In general, Dex‐TCNP provided greater protection from organ injury than free Dex, suggesting that TCNP‐mediated delivery enhances the therapeutic efficacy and tissue‐protective capacity of Dex.

**FIGURE 6:**
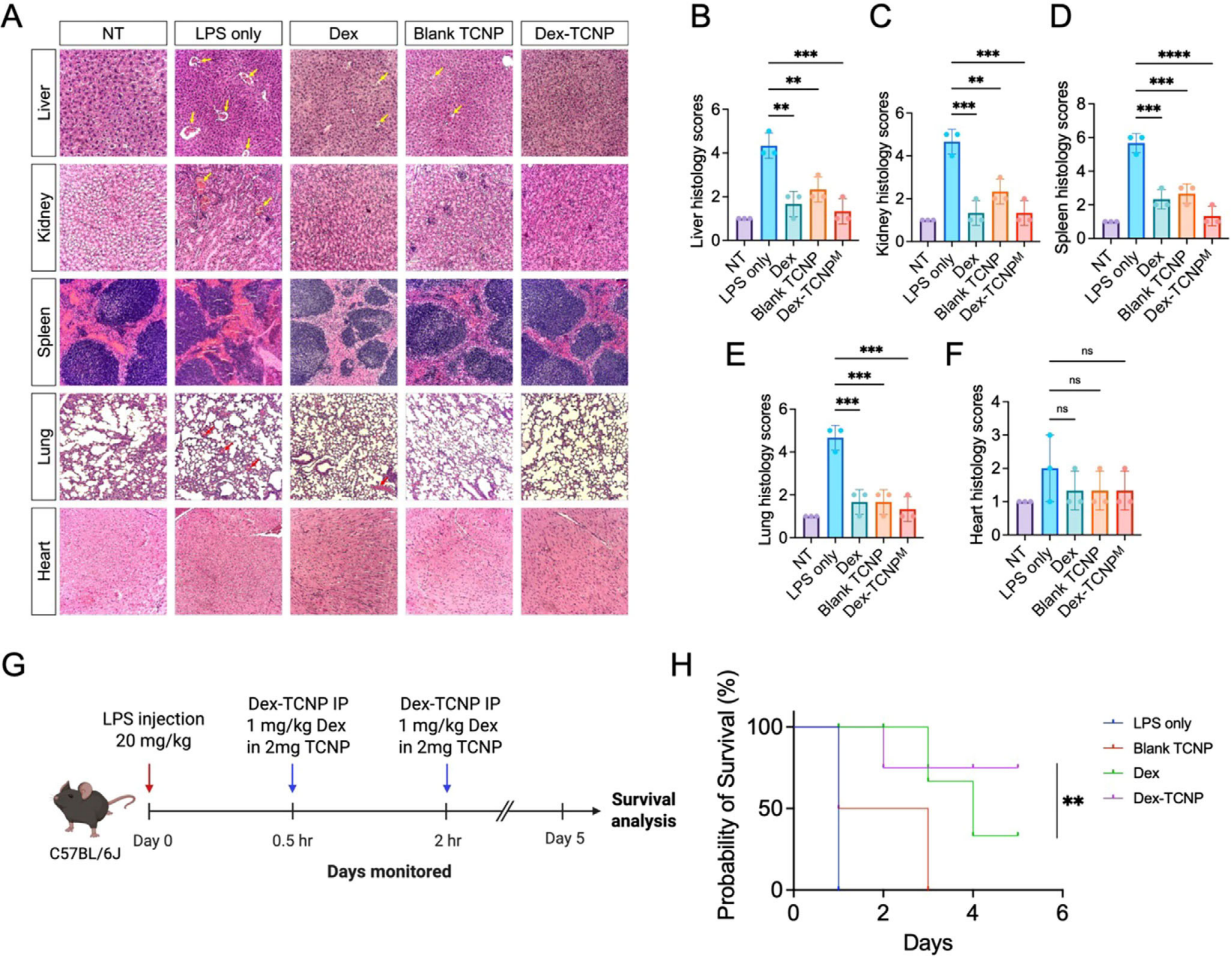
Histological examination of major organs and survival study using LPS‐induced endotoxemia mouse model. (A) Hematoxylin and eosin (H&E) staining of liver, kidney, spleen, lung and heart collected from Figure 5. (B–F) Quantification of H&E staining. Level of injury score were calculated. Data are expressed as mean ± SD (*n* = 3) **p < 0.01, ***p < 0.001, ****p < 0.0001. (G) Schematic depicting experimental design to evaluate survival in a lethal LPS‐induced endotoxemia model. Created in BioRender. Sung, J. (2026) https://BioRender.com/l63d302. (H) Kaplan‐Meier curves and a log‐rank test was performed to compare the survival rates between treatment groups over 5 days. **p < 0.01.

Finally, survival was evaluated in a lethal LPS‐induced endotoxemia rescue model. Mice were first challenged with 20 mg/kg LPS and subsequently treated intraperitoneally with two doses of Dex‐TCNP, TCNP, or Dex (Figure 6G). Dex‐TCNP treatment significantly improved survival, with 80% of mice surviving to day 5 compared to 40% survival in the free Dex group (Figure 6H). Blank TCNP showed meaningful partial protection, extending survival beyond the LPS only group where all mice succumbed within 24 h, though without sustained long‐term survival enhancement. This partial efficacy is consistent with the platform's cytokine sequestering activity in the absence of intracellular drug delivery and establishes that the TCNP scaffold itself is therapeutically active. In contrast, Dex‐TCNP achieved 80% survival at day 5, demonstrating that programmable drug loading is required to convert the activity of TCNP into durable therapeutic benefit. Mechanistically, sustained elevation of IL‐6 strongly correlates with disease severity, organ failure and mortality in both experimental and clinical settings [35], whereas timely induction of IL‐10 is critical for limiting excessive inflammatory damages and promoting resolution of systemic inflammation [36]. Clinical and pre‐clinical studies have shown that coordinated IL‐10 responses, particularly in the context for reduced pro‐inflammatory signaling, are associated with improved immune homeostasis and survival [37]. Accordingly, the coupled reduction of IL‐6 and enhancement of IL‐10 observed with Dex‐TCNP reflects a therapeutically favorable shift toward a pro‐resolving immune state. Taken together, these findings demonstrate that TCNP‐mediated delivery of Dex is significantly more effective than free drug administration, improving survival, while reprogramming immune responses toward resolution. These results highlight the potential of TCNP as a versatile nanotherapeutic platform for improving outcomes in acute inflammatory diseases such as sepsis.

## 3 Discussion

The cytokine storm and its downstream sequelae including multi‐organ failure and sepsis‐induced immunosuppression remain leading cause of critical illness mortality, yet no therapeutic platform has successfully addressed the polycytokine, multi‐pathway nature of this pathology [38]. Current standard‐of‐care approaches, including corticosteroid and cytokine‐targeting biologics, are fundamentally limited by broad immunosuppression, antigen specificity, systemic toxicity and inability to restore immune homeostasis rather than simply attenuating it [39, 40, 41]. While our previously reported MDNP demonstrated compelling activity against these limitations through multimodal protein corona‐mediated cytokine sequestration, its clinical translation remains constrained by low yield and batch‐to‐batch variability. Guided by MDNP lipidomic analysis, TCNP was developed at the optimal ratio of 75:25 TG:Cer (Figure 2A) to overcome these barriers by reverse‐engineering the functional lipid identity of MDNP into a simplified, reproducible and scalable synthetic platform that preserves the inherited cytokine‐scavenging bioactivity of its natural composition, while enabling programmable drug delivery (Table 1). This design philosophy uniquely places TCNP apart from the broader landscape of anti‐inflammatory nanoparticles which are generally conceived as drug carriers first with immunomodulatory function being a consequence of the delivered payload rather than an intrinsic material property. TCNP inverts this paradigm through its primary identity being a cytokine sequestering nanoparticle via multimodal protein corona formation with drug encapsulation and delivery features enabling an additional engineered layer built upon that foundation.

Several cytokine scavenging nanoplatforms have been reported as alternative to conventional anti‐inflammatory agents and biologics, including cell membrane‐coated nanosponges [42], antibody‐conjugated nanoparticles [43], aptamer‐functionalized systems [44] and magnetic nanoparticles for extracorporeal blood purification [45], demonstrating proof‐of‐concept cytokine adsorption. However, these platforms typically require complex post‐synthetic surface functionalization to achieve cytokine binding, lack integrated drug delivery capacity and have limited in vivo efficacy data in severe inflammation models. Extracorporeal hemadsorption devices such as CytoSorb represent the most clinically advanced cytokine removal strategy yet require continuous vascular access and do not address the intracellular inflammatory signaling that perpetuate cytokine production [46]. TCNP requires no complex surface modification to access its intrinsic cytokine sequestering ability and can be developed to simultaneously deliver synergistic anti‐inflammatory drugs to repress pre‐established cytokine storm, while stopping further pro‐inflammatory cytokine transcription through intracellular drug delivery.

The functional advantage of the dual mechanism design of TCNP was demonstrated across multiple levels of biological resolution. At the extracellular level, TCNP adsorbed IL‐6 and TNF‐α in a concentration‐dependent manner through multimodal protein corona formation (Figure 2E–G) and SDS‐PAGE revealing a conserved protein corona fingerprint shared with MDNP (Figure 2H). This conservation of corona composition strongly suggests that the TG‐Cer lipid surface is the primary structural determinant of cytokine‐binding capacity, validating the reverse‐engineering strategy.

At intracellular level, time‐dependent internalization of TCNP by BMDM (Figure 3E,F) combined with the sustained release of Dex from TCNP (Figure 3D) enabled intracellular glucocorticoid receptor engagement, directly accounting for the enhanced NF‐κΒ suppression (Figure 3J) and cytokine reduction observed with Dex‐TCNP relative to concentration‐matched free Dex (Figure 3H,I). Importantly, the transcriptional response to Dex‐TCNP was not a simple amplification of free Dex activity, but a qualitatively distinct immunomodulatory reprogramming event. While free Dex broadly suppressed canonical inflammatory genes (Figure 4B,C), Dex‐TCNP further and selectively downregulated Il6, while uniquely inducing *Il10*, *Creb1*, and *Areg* (Figure 4B,D) which are associated with NF‐κB antagonism [47], macrophage immune tolerance [31] and epithelial tissue repair [48], respectively. Noticeable immunomodulatory activity of blank TCNP was also observed, as evidenced by its distinct transcriptional signature including suppression of *Arg1*, *Hif1a*, *il6 and Nos2* alongside upregulation of *Hmgn1*, *Myc* and *Tgfbr1*, further demonstrating that the TCNP itself engages immune regulatory programs upon internalization, reinforcing that its therapeutic bioactivity may extends beyond protein corona‐mediated cytokine capture effects (Figure 4E).

The pathway analysis further conceptualized the transcriptional response to Dex‐TCNP within the established immunomodulation framework. The enriched pathways spanning cytokine‐mediated signaling pathways, negative regulation of the NF‐κB cascade, chemokine signaling, and apoptotic regulation collectively reflect a coordinated shift away from pro‐inflammatory amplification toward immune resolution rather than broad suppression. The enrichment of negative NF‐κB regulatory pathways with Toll‐like receptor signaling pathways suggests that Dex‐TCNP engages multiple layers of innate immune regulation simultaneously [49] that may not be observed with free Dex (Figure 4H). The IL‐6‐centered gene network visualized the tight connectivity among downregulated nodes‐including *Il6*, *Tnfa*, *Nos2*, *Ptgs2*, and *Hif1a* indicating that Dex‐TCNP does not suppress individual inflammatory genes but rather dismantles a coherent inflammatory program. The concurrent upregulation of *Il10*, *Tgfbr1* and *Cd55* further supports a model of active immunological rebalancing highlighting that the therapeutic mechanism of Dex‐TCNP operated at the level of immune network architecture (Figure 4I).

These molecular and cellular effects translated directly into in vivo therapeutic benefit. In the LPS endotoxemia model, Dex‐TCNP was the only treatment to achieve the coordinated cytokine response most predictive of survival, simultaneously suppressing IL‐6 and TNF‐α with significant IL‐10 induction (Figure 5E–G). This cytokine profile was reflected in near‐baseline histological injury scores across liver, kidney, spleen, lung and heart (Figure 6B–F), and supported by reduced ALT and BUN levels indicating attenuated hepatic [50] and renal [51] injury (Figure S5A,G). Most importantly, Dex‐TCNP achieved 80% survival in a lethal endotoxemia model compared to 40% for free Dex (Figure 6H). The survival data provide definitive in vivo validation of the dual mechanism design principle.

The preferential accumulation of TCNP in the liver and kidney (Figure 5B,C) is consistent with the multi‐organ protective effects of Dex‐TCNP. This distribution pattern suggests a dual mechanism in which targeted organ deposition facilitates local immunomodulation, while also driving systemic anti‐inflammatory effects that extends to distant organs. Systemic cytokine sequestration via protein corona formation acts within the circulation to broadly attenuate pathological cytokine levels independently of direct nanoparticle accumulation in target tissues. Thereby reducing organ injury across the liver, kidney, spleen, lung and heart.

An important consideration in the translational development of any bioactive protein corona‐forming nanoparticles is the potential for off‐target sequestration of non‐pathological plasma proteins including protective cytokines, growth factors, complement components and regulatory mediators. While the broad adsorptive capacity of the TCNP corona is central to its therapeutic mechanisms, the selectivity of this interaction under pathological versus homeostatic conditions warrants careful evaluation. Critically, the therapeutic context of TCNP administration is acute cytokine storm, a pathological state characterized by massively elevated concentration of pro‐inflammatory mediators that dominate the protein binding landscape and are likely to outcompete homeostatic proteins for corona occupancy at the administered dose. This concentration‐driven preferential enrichment of pathological cytokine is consistent with the observed IL‐10 induction in vivo (Figure 5G), suggesting that regulatory cytokine signaling remains intact following TCNP treatment rather than being broadly suppressed. Furthermore, the normal organ histology (Figure 6A–F) and blood biochemistry (Figure S5) observed in treated animals provide in vivo evidence against gross perturbation of immune homeostasis or complement‐mediated organ injury at the doses tested. Nevertheless, the full spectrum of corona‐associated proteins under homeostatic conditions, the potential consequences of repeated administration on host defense, and the long‐term data of TCNP‐protein complexes following hepatic and renal accumulation remain important considerations for future preclinical safety evaluation as the platform advances toward translational development.

While this work established strong proof‐of‐concept for TCNP in acute LPS‐driven endotoxemia, evaluation in polymicrobial sepsis models and immunotherapy‐associated cytokine release syndrome (CRS) will be meaningful to define the full breadth of therapeutic applicability. The conserved SDS‐PAGE corona fingerprint shared between TCNP and MDNP, together with previously reported proteomic characterization of native MDNP demonstrating inflammatory cytokine enrichment, supports the mechanistic inference that TCNP inherits and analogues sequestration capacity though its shared lipid surface identity. To further substantiate the sequestration mechanisms, direct quantification of IL‐6 and TNF‐α across paired supernatant and pellet fractions following incubation of TCNP with LPS‐stimulated conditioned media confirmed the inference where cytokine levels were markedly depleted in the post‐incubation supernatant, while the dissociated pellet fraction demonstrated corresponding cytokine enrichment with mass balance between fractions approximating total input (Figure S3). This partitioning provides cytokine‐level confirmation that TCNP actively sequesters multiple inflammatory mediators on its surface, distinguishing true corona‐mediated capture from non‐specific depletion onto its surface or assay interference. The recovery of the TCNP‐cytokine pellet did not fully account for the depleted cytokines, consistent with the known difficulty of achieving complete protein recovery from a nanoparticle surface, particularly with milder detergent Triton X‐100, used in the test to preserve cytokine epitope integrity are inherently less exhaustive than denaturing agents like SDS [52]. Additionally, residual or incompletely dissociated protein corona has been shown to alter antibody binding affinity in sandwich immunoassays which may further limit detection of corona‐associated cytokine by ELISA [52]. Nonetheless, this partial recovery is therefore consistent with a true surface‐sequestration mechanism, reflecting the inherent limitations of mild detergent‐based elution rather than degradation or loss of the sequestered cytokines.

## 4 Conclusions

This work establishes TCNP as a reverse‐engineered lipid nanoparticle platform that mitigates severe systemic inflammation through dual mechanisms of inherited cytokine sequestration and programmable drug delivery. Guided by lipidomic profiling of native MDNP, TCNP formulation recapitulated key physicochemical and immunomodulatory features, while enabling reproducible and scalable formulation. In vitro studies demonstrated enhanced anti‐inflammatory activity of Dex‐loaded TCNP, including suppression of IL‐6 and TNF‐α production and inhibition of NF‐κB signaling. Consistent with these functional effects, transcriptional profiling revealed IL‐6‐centric reprogramming characterized by suppression of inflammatory signaling pathways and induction of the regulatory cytokine IL‐10. Importantly, these molecular and cellular effects translated into in vivo therapeutic efficacy, where Dex‐TCNP reduced systemic inflammation, improved organ protection, and significantly enhanced survival in a lethal murine endotoxemia model. In conclusion, dual function cytokine sequestering and drug carrying TCNP represents an innovative immunomodulatory nanotherapeutic strategy that addresses both extracellular cytokine burden and intracellular inflammatory signaling that conventional therapies fail to resolve simultaneously, offering a translatable nanoplatform for cytokine storm‐driven inflammatory diseases.

## 5 Experimental Section

### 5.1 Synthesis and Characterization of Triglycerides‐Ceramide Lipid Nanoparticles (TCNP)

TCNP were prepared using the thin film hydration method. TG (medium chain triglycerides; Spectrum Chemical, New Brunswick, NJ) and ceramides (Cayman Chemical, Ann Arbor, MI) were dissolved in a High‐Performance Liquid Chromatography (HPLC) grade ethanol at different molar ratios (75:25, 50:50, and 25:75, TG:Ceramide). The solvent mixture was mixed on stir plate for an hour and was evaporated under reduced pressure in a rotary evaporator (Buchi R‐300, Switzerland) at 40°C, 300 mbar, and 200 rpm until a uniform thin film was formed on the flask wall for up to 1 h. The lipid film was rehydrated with pre‐warmed PBS (pH 7.4) with gentle shaking and rotating in bath sonicator, yielding multilamellar vesicles. To reduce particle size and achieve homogeneity, suspensions were subjected to extrusion at 40°C and 75°C through polycarbonate membranes (100 nm, Avanti Polar Lipids, Alabaster, AL) using a mini extruder (Avanti Polar Lipids). The fully synthesized nanoparticles were stored at 4°C. The size and zeta potential of TCNP were measured by dynamic light scattering (DLS) using a Zetasizer Nano ZSP (Malvern Panalytical, UK). Transmission electron microscopy (TEM) images of TCNP were acquired using a formvar‐coated copper grid and staining using 1% uranyl acetate.

### 5.2 Storage Stability Assessment

TCNP were formulated at 75:25 ratio and extruded at 40°C. Freshly prepared TCNP were aliquoted and stored either at room temperature or 4°C for up to 10 days in PBS. At designated time points, particle hydrodynamic diameter and zeta potential were quantified by dynamic light scattering to monitor colloidal stability over time.

### 5.3 Ex Vivo Concentration‐Dependent Pro‐Inflammatory Cytokine Sequestration Assay

TCNP suspensions were prepared at concentrations of 50, 100, 200, and 300 µg/mL in PBS. Each preparation was combined with 100 µL of diluted LPS‐stimulated mouse plasma in ultracentrifuge tubes. Samples were incubated at 37°C for 8 hrs and subsequently centrifuged at 13 000 × g for 10 min to pellet cytokine‐bound TCNP. Supernatants were collected, and residual IL‐6 and TNF‐α concentrations were determined using ELISA (BioLegend).

### 5.4 Qualitative Assessment of TCNP Protein Corona Formation

To assess protein corona formation on TCNP, they were incubated with conditioned cell culture media collected from LPS‐treated bone marrow‐derived macrophages (BMDM). Briefly, TCNP were mixed with the BMDM supernatant and incubated for 4 h at 37°C under gentle agitation. After incubation, TCNP‐protein complexes were isolated by centrifugation at 13 000 × g for 20 min at 4°C. The resulting pellet, containing protein‐bound TCNP, was washed three times with ice‐cold 1× PBS to remove unbound proteins. Subsequently, protein coronas were detached from the TCNP surface by resuspending the pellets in 2× Laemmli sample buffer (Bio‐Rad) containing 5% β‐mercaptoethanol, followed by heating at 95°C for 5 min. Samples were then separated on a 4%–15% SDS‐PAGE gel under reducing conditions. Gels were stained with Coomassie Brilliant Blue R‐250 (Bio‐Rad) for 1 h at room temperature and destained using 40% methanol and 10% acetic acid until protein bands were clearly visible. Protein banding patterns were imaged using a Chemidoc MP imaging system (Thermo Fisher), providing a qualitative profile of the TCNP‐associated protein corona. Changes in particle size before and after corona formation were evaluated by incubating TCNP with LPS‐stimulated culture media and analyzing via dynamic light scattering (DLS).

### 5.5 Quantitative Assessment of Physical Association of Corona‐Associated Pro‐Inflammatory Cytokines on TCNP

To confirm that the reduction in cytokine levels reflected physical sequestration onto the TCNP surface rather than degradation or non‐specific loss, paired supernatant and corona‐dissociated pellet fractions were quantified for IL‐6 and TNF‐α. TCNP were incubated with LPS‐stimulated BMDM‐conditioned media for 4 h at 37°C under gentle agitation. Samples were centrifuged at 13 000 x g for 20 min at 4°C; the resulting supernatant was collected for direct ELISA quantification of IL‐6 and TNF‐α. Pellets were washed twice to remove unbound cytokine, then resuspended in PBS containing 0.2% Triton X‐100 to dissociate the protein corona [53]. Lysates were clarified by centrifugation at 1000 x g for 5 min) and the resulting supernatant was assayed by ELISA (BioLegend). TCNP incubated in PBS alone and processed identically served as a control.

### 5.6 Evaluating Encapsulation Efficiency and Drug Release Profile

Three different doses of Dex encapsulated TCNP (Dex‐TCNP^L^, Dex‐TCNP^M^, and Dex‐TCNP^H^) were prepared and characterized for drug encapsulation efficiency (EE) using UV–vis spectrophotometry. Briefly, an aliquot of each nanoparticle suspension was centrifuged at 130 000 x g for 1 h to separate free (unencapsulated) Dex. The supernatant containing free drug was collected and its absorbance was measured at 240 nm, the characteristic absorbance peak of the drug. A calibration curve of Dex in PBS was used to determine the concentration of free drug. Encapsulation efficiency was calculated using the formula below.

$$EE\;\left(\%\right)=\left(1-\frac{\mathrm{amount}\;\mathrm{of}\;\mathrm{free}\;\mathrm{drug}}{\mathrm{total}\;\mathrm{drug}\;\mathrm{loaded}}\right)\;\times 100$$

For drug release profiling, each Dex‐loaded nanoparticle formulation was suspended in PBS and incubated at 37°C under gentle shaking. At predetermined time points (0, 2, 4, 8, 12, 24 h), samples were ultracentrifuged at 150,000 x g for 1 h to separate released drug in the supernatant. The supernatant absorbance at 240 nm was measured by UV–vis spectrophotometry, and the cumulative percent drug release was calculated relative to the total drug encapsulated. Drug release profile of Dex‐TCNP were plotted as cumulative drug release (%) versus time to compare their release kinetics.

### 5.7 In Vitro Cellular Internalization

BMDM were plated on Falcon culture slides at a density of 0.5 × 10^5^ cells per well and allowed to adhere overnight. TCNP were fluorescently labeled with Cy5.5 dye by incubating under gentle agitation for 30 min protected from light, followed by centrifugation at 13 000 × g for 20 min to remove unincorporated dye. Subsequently, 50 µL of Cy5.5‐labeled TCNP were added to 0.5 mL culture medium and incubated with cells for 0, 4, or 8 hrs, with untreated cells serving as the 0 h control. At each time point, cells were washed three times with PBS, fixed with 4% paraformaldehyde for 10 min, and dehydrated in acetone at −20°C for 5 min. After blocking with 1% BSA in PBS for 30 min, cells were stained with FITC‐conjugated phalloidin (1:50 dilution) for 30 min to visualize F‐actin. Following two PBS washes, coverslips were mounted with DAPI‐containing mounting medium after carefully removing the plastic chambers. Fluorescence images were acquired using an Olympus fluorescence microscope equipped with a Hamamatsu ORCA‐03G digital camera and analyzed with Nikon software.

To quantify time‐dependent cellular uptake, flow cytometry was performed using BMDM plated at a density of 0.5 × 10^5^ cells per well and treated with Cy5.5‐labeled TCNP for 0, 2, 4, or 8 h. At each time point, cells were harvested with versene and resuspended in MACS buffer (PBS pH 7.2 supplemented with 1% FBS and 0.4% 0.5 M EDT (Quality Biological, Gaithersburg, MD) and transferred to the flow cytometry tubes. Cells were first incubated with an FcR blocking reagent (anti‐CD16/32, Biolegend, San Diego, CA) and subsequently stained with surface marker antibodies: fixable green, CD11b, and F4/80) to confirm viability and BMDM identity. Sample data was acquired on Cytek Aurora 3 (Fremont, CA). FCS Express 7 Flow cytometry De Novo Software was used to process the data.

### 5.8 In Vitro Pro‐Inflammatory Cytokine Removal and Effects on NF‐κB Activity

BMDM were stimulated with 100 ng/mL lipopolysaccharide (LPS) for 4 hrs. Subsequently, free Dex or Dex‐loaded TCNP (at 0.8, 8, or 80 ng/mg Dex‐TCNP, w/w) was added, followed by incubation for 8 h. After incubation, culture supernatants were collected, and levels of IL‐6, TNF‐α were quantified using enzyme‐linked immunosorbent assay (ELISA; BioLegend, San Diego, CA). RAW‐Blue macrophage reporter cell, which secretes SEAP in proportion to NF‐𝜅B activation, were seeded at 1.0 × 10^5^ per well. After 4 h stimulation with LPS, cells were treated with Free Dex, Blank TCNP, or Dex‐TCNP^M^ for 8 h. Free Dex was concentration matched against Dex in Dex‐TCNP^M^ and TCNP was concentration matched against TCNP in Dex‐TCNP^M^. The cell culture supernatant was then transferred to a flat‐bottom 96‐well plate and mixed with QUANTI‐Blue reagent. Following a 1‐hr incubation at 37°C, SEAP activity used as a surrogate readout of NF‐κΒ activation was measured using a spectrophotometer at 620–655 nm.

### 5.9 NanoString nCounter Gene Expression

Total RNA was extracted from LPS‐stimulated BMDM treated with free Dex, blank TCNP or Dex‐TCNP using the RNeasy Mini Kit (Qiagen) according to manufacturer's instructions. RNA quality and concentration were assessed by Agilent Bioanalyzer. For each sample, 100 ng of total RNA was used in the NanoString nCounter assay with the Mouse Inflammation Panel (NanoString Technologies), which profiles the expression of 254 inflammation‐related genes and 6 reference genes. Samples were hybridized with reporter and capture probes at 65°C overnight. Hybridized samples were then processed and immobilized on cartridges using the nCounter Prep Station, followed by scanning on the nCounter Digital Analyzer. Raw data (RCC files) were imported into nSolver Analysis Software (Version 4.0, NanoString Technologies) for quality assessment and normalization, using the geometric mean of housekeeping genes. Differential gene expression analysis and data visualization were performed using the nCounter Advanced Analysis software. Venn diagram was generated by GeneVenn to visualize overlaps and treatment‐specific gene regulation among the groups, enabling identification of uniquely and commonly regulated gene sets. The enrichment analysis was performed using Metascape showing signaling pathways identified based on default statistical parameters. The results were ranked by ‐log_10_ (p‐value) and visualized as horizontal bar plots. Gene network analysis was performed using NetworkX, with co‐expression relationships defined by Pearson correlation of normalized NanoString expression values.

### 5.10 Cell Culture

BMDM were generated using a previously described protocol. Briefly, tibias and femurs were harvested from C57BL/6J mice after removal of muscle and connective tissue. Bone marrow was flushed with RPMI medium supplemented with L‐glutamine, penicillin (100 U/mL), streptomycin (100 µg/mL), 10% heat‐inactivated fetal bovine serum (FBS), and 20% L929 cell‐conditioned media. The marrow was collected into 10 cm petri dishes and filtered before plating in uncoated 10 cm culture plates. Cells were maintained at 37°C and 5% CO_2_ with medium changes every 3 days. By days 8–10, BMDM were detached using Versene (Gibco) and counted for viability using trypan blue and an EVE Automated Cell Counter (NanoEntek). RAW‐blue cells were also expanded to confluency in 75 cm^2^ flasks at matching conditions using DMEM containing 10% heat‐inactivated FBS, penicillin (100 U/mL), and streptomycin (100 U/mL).

### 5.11 Mice

Male C57BL/6 mice (6–8 weeks old) procured from Jackson Laboratory (Bar Harbor, ME) and acclimatized under controlled housing conditions including ambient temperature, 55% relative humidity, 12 h light/dark cycle. For endotoxemia induction, mice received an intraperitoneal (IP) injection of LPS at 5 mg/kg, followed by two intraperitoneal doses of TCNP (2 mg IP each) at 30 min and 2 h after challenge. For experimental treatments, mice were administered Dex‐TCNP (1 mg/kg Dex in 2 mg TCNP/dose, IP) at 30 min and 2 h after LPS challenge. Dosing regimens were selected based on previously reported tolerability of multiple nanoparticle administrations. All procedures were reviewed and approved by the University of Maryland, Baltimore Institutional Animal Care and Use Committee (IACUC) and adhered to ARRIVE guidelines (IACUC Protocol # AUP‐00000975).

### 5.12 Cytotoxicity Assay

BMDMs were differentiated for 7 days then seeded in flat bottom 96‐well plates at 1 × 10^5^ cells/well in DMEM. After overnight adherence, cells were treated with Dex and Dex‐TCNP (4, 40 and 400 ng/mL) and 200 ug/mL TCNP in assay medium. At the end point (24 h), culture plates were gently centrifuged at 200 x g, 5 min, and 50 µL of clarified supernatants from each well was transferred to a fresh 96‐well plate. LDH activity was quantified using a commercial LDH kit according to the manufacturer's protocol: 50 µL of LDH reaction mixture was added to each supernatant, incubated for 30 min at room temperature protected from light, and stop solution was added before measuring absorbance at 490 nm with a 680 nm reference. 1% Triton X‐100 was used as a positive control.

### 5.13 In Vivo Biodistribution and Anti‐Inflammatory Effect Following LPS Challenge

To assess tissue distribution of TCNP in vivo, Cy5.5 near‐infrared fluorescent dye was incorporated within TCNP prior to administration. LPS‐challenged mice (5 mg/kg) received two intraperitoneal injections of Cy5.5‐TCNP (2 mg/dose) at 30 min and 2 h post‐challenge. At the 4 h endpoint, animals were euthanized, and blood was obtained by cardiac puncture. Major organs including liver, lungs, spleen, heart, and kidneys were surgically excised, and fluorescence signal was captured using IVIS with laser excitation at 675 nm and emission collected at 720 nm to map nanoparticle biodistribution. Collected blood was processed into plasma by centrifugation at 1000 × g for 20 min at 4°C using microtainer capillary collection tubes (BD Diagnostics, Franklin Lakes, NJ). Plasma cytokine profiles were determined using a multiplexed Luminex MAGPIX platform (Luminex, Austin, TX) with a 7‐analyte panel targeting murine IL‐6, TNF‐α, MCP‐1, IL‐10, IL‐1β, IFN‐β, and GROα. Systemic organ function was further assessed through a comprehensive blood chemistry panel measuring ALT/SGPT, AST/SGOT, creatinine, BUN, total protein, globulin, and albumin, performed by VRL Animal Health Diagnostics (Gaithersburg, MD). For histopathological evaluation, organs were fixed immediately in 4% formalin upon harvest, embedded in paraffin, and sectioned for hematoxylin and eosin (H&E) staining performed by the Pathology Biorepository Shared Services Core at the University of Maryland, Baltimore. Tissue injury was scored by a blinded assessor using a semi‐quantitative 6‐point scale: 0 indicating no detectable damage, and 5 representing extensive necrosis affecting more than 75% of the tissue section.

### 5.14 In Vivo Systemic Inflammation Survival Study

The in vivo survival study was carried out using a representative severe systemic inflammation model: a lethal LPS‐induced endotoxemia mouse model. C57BL/6J mice were challenged with 20 mg/kg LPS and two doses of 1 mg/kg Dex within 2 mg TCNP administered via IP injection at 0.5 and 2 h time point. Survival for each model was evaluated over the course of 5 days. The total TCNP administered was selected based on in vitro concentration‐response data establishing 200 µg/mL as the optimal concentration for sequestration, scaled to approximate an effective circulating concentration following IP administration. Also, it is precedent from our previously established study in equivalent endotoxemia model with MDNP [23].

### 5.15 Statistical Analysis

Data analysis was performed using GraphPad Prism Software 10.4.1 (San Diego, CA). One‐way ANOVA and t test for unpaired data were employed to evaluate statistical significance along with Tukey's multiple comparison test. Kaplan‐Meier survival curve and statistical significance of mouse survival were determined with a long‐rank (Mantel‐Cox) X2 test. Significant differences were indicated as *p < 0.05, **p < 0.01, ***p < 0.001, and ****p < 0.0001, related to control unless otherwise stated.

### 5.16 AI Usage Statement

Claude (Anthropic) was used to assist with grammar correction and improvement of sentence structure. The authors reviewed and edited all content and take full responsibility for the manuscript. No AI tools were used for data analysis or figure generation.

## Author Contributions

J.J.S., conceptualization, formal analysis, investigation, methodology, visualization, and writing – original draft, review and editing; G.Y.D., investigation and methodology, and R.M.P., conceptualization, funding acquisition, methodology, resources, supervision, and writing – original draft, review and editing.

## Funding

This work was supported by the National Institute of General Medical Sciences (R35GM142752) and National Institute for Allergy and Infectious Diseases (R01AI192405 and R21AI187784) of the National Institutes of Health under award number awarded to R.M.P. J.J.S was supported by the University of Maryland Strategic Partnership: MPowering the State Early Scholars Investment Fund Award. This publication was supported by funds through the Maryland Department of Health's Cigarette Restitution Fund Program and the National Cancer Institute—Cancer Center Support Grant (CCSG)—P30CA134274; University of Maryland School of Medicine's Center for Translational Research in Imaging—Baltimore, Maryland; University of Maryland School of Medicine Genomics Core—Baltimore, Maryland and University of Maryland School of Medicine's and School of Dentistry's Electron Microscopy Core—Baltimore, Maryland. BioRender was used for preparing graphics. The content is solely the responsibility of the authors and does not necessarily represent the official view of the National Institutes of Health.

## Conflicts of Interest

The authors declare no conflicts of interest.

## Supporting information




**Supporting File**: smll74914‐sup‐0001‐SuppMat.pdf.

## Data Availability

Experimental details and additional data are available in Method section and in the ESI. Any other supporting this article are available from the corresponding authors upon request.
