# Supplementary material for: Reverse‐Engineered Lipid Nanoparticles Harness Protein Corona Formation for Cytokine Sequestration and Combination Immunomodulatory Therapy
Source: Small. 2026 Jul 29;22(55):e74914. doi: 10.1002/smll.74914 (PMC13630403; doi:10.1002/smll.74914)
Supplement: Supplementary file 1 [file SMLL-22-e74914-s001.pdf]

Supporting Information

Reverse-Engineered Lipid Nanoparticles Harness Protein Corona Formation for Cytokine Sequestration and Combination Immunomodulatory Therapy

Junsik J. Sung[1], Gargi Yogesh Digholkar[1], Ryan M. Pearson[1,2,3*]

1. Department of Pharmaceutical Sciences, University of Maryland School of Pharmacy, 20 N. Pine Street, Baltimore, MD 21201, USA

2. Department of Microbiology and Immunology, University of Maryland School of Medicine, 685 W. Baltimore Street, Baltimore, MD 21201, USA

3. Marlene and Stewart Greenebaum Comprehensive Cancer Center, University of Maryland School of Medicine, 22 S. Greene Street, Baltimore, MD 21201, USA

[*]Address correspondence to:
Ryan M. Pearson, Ph.D.
Associate Professor, Department of Pharmaceutical Sciences
Director, Bio- and Nano-Technology Center
University of Maryland School of Pharmacy
20 N. Pine Street
N525 Pharmacy Hall
Baltimore, MD 21201, US
Phone: 410-706-3257
Email: rpearson@rx.umaryland.edu

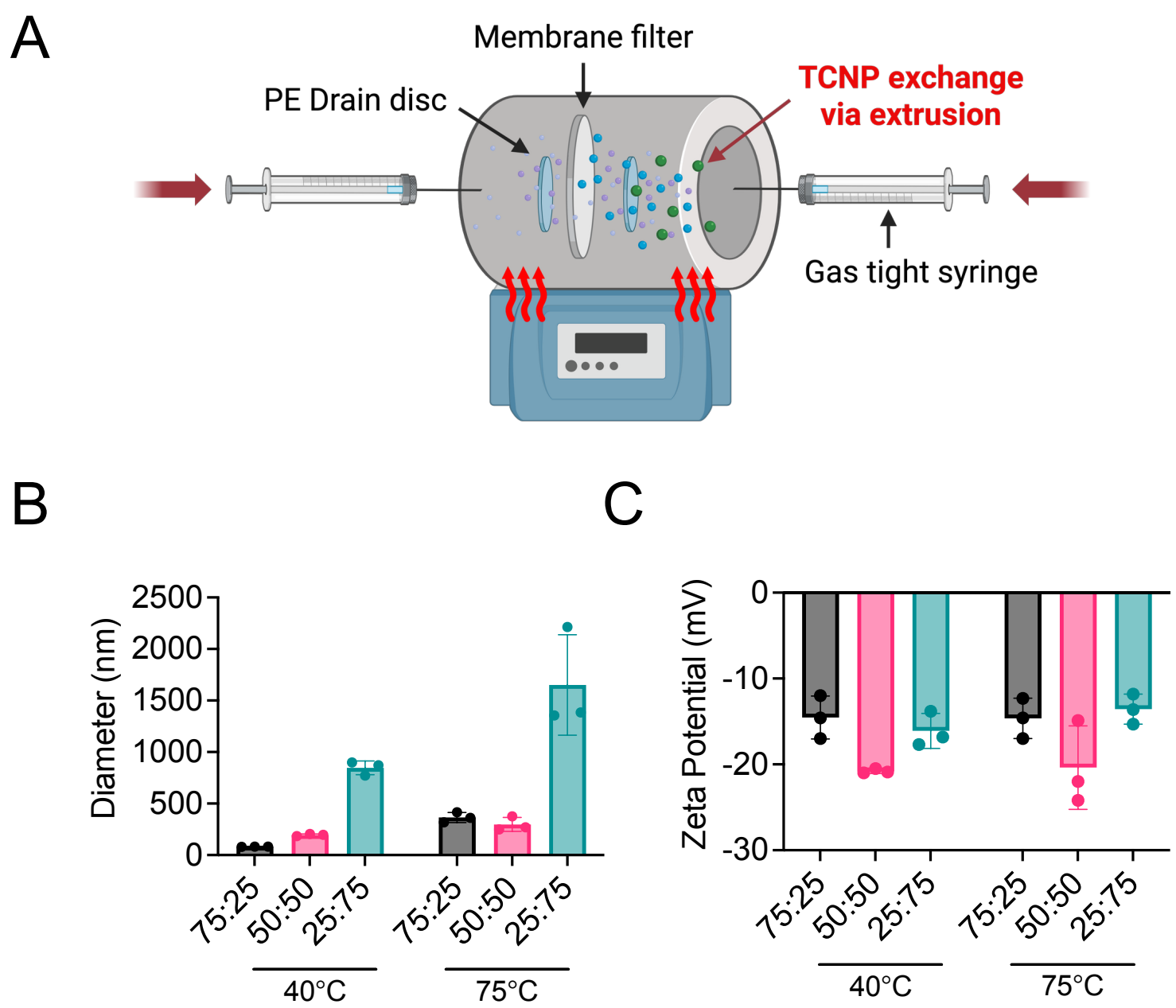

Figure S1. Optimization of TCNP formulation via mini extruder system (A) Schematic illustration of the TCNP exchanged using mini extruder system. TCNP were formulated at varying ratios and subjected to extrusion at two different temperatures to determine optimal physicochemical properties. Created in BioRender. Sung, J. (2026) https://BioRender.com/8dl1qnw. Physicochemical characterization of TCNP formulations by dynamic light scattering (DLS). (B) Hydrodynamic diameter and (C) zeta potential was measured across all tested conditions. A lipid ratio of 75:25 extruded at 40ºC yielded the most favorable particle size and surface charge profile and was selected for subsequent studies.

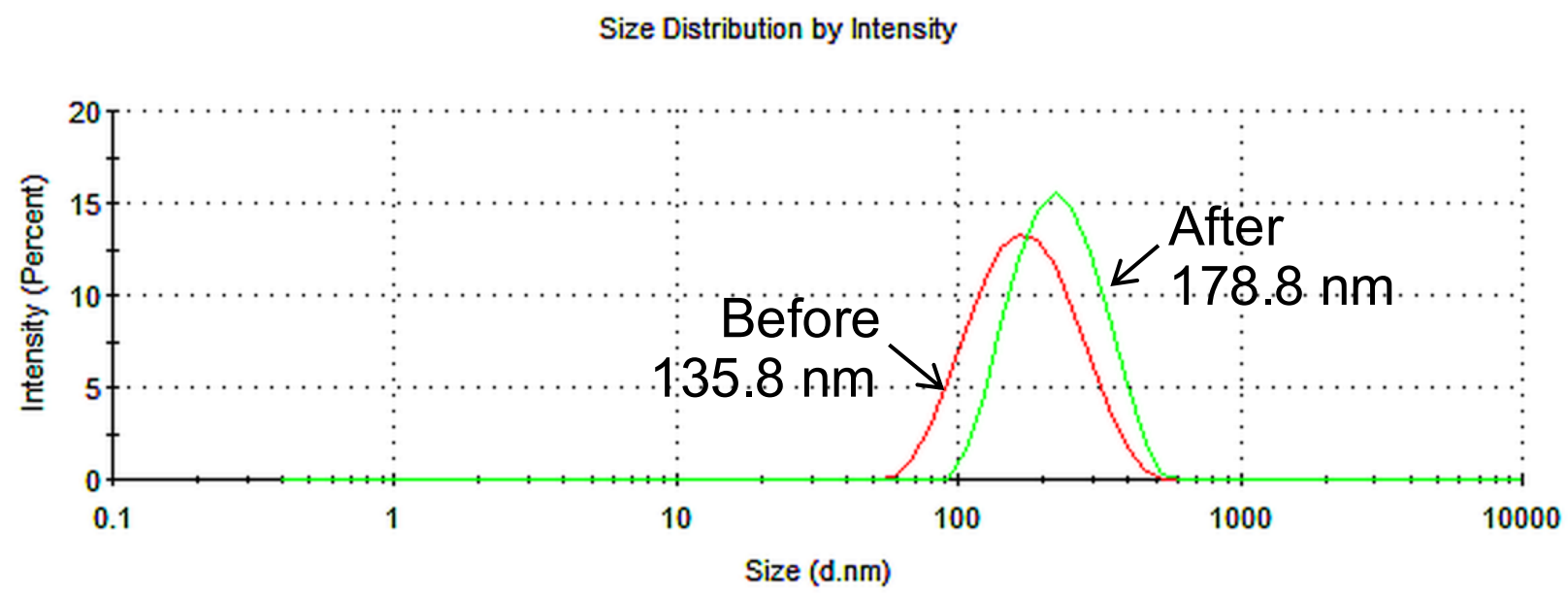

Figure S2. The hydrodynamic diameter of TCNP increased from 135.8 nm to 178.8 nm following incubation in conditioned media collected from LPS-stimulated BMDM, indicative of protein corona formation on the nanoparticle surface.

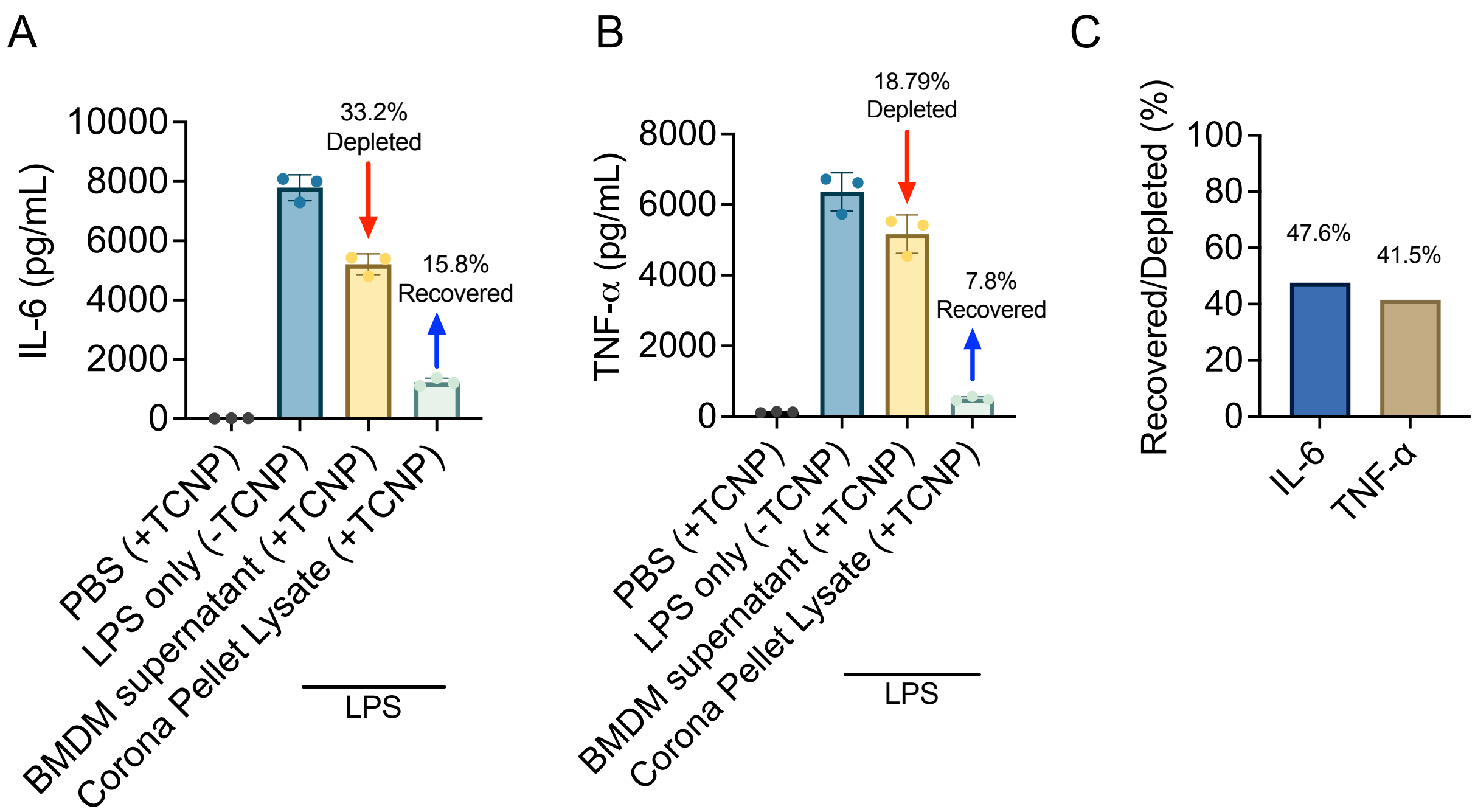

Figure S3. Paired supernatant/pellet quantification of physical sequestration of pro-inflammatory cytokines. TCNP were incubated with LPS-stimulated BMDM-conditioned media and (A) IL-6 and (B) TNF-α were quantified by ELISA in the post-incubation supernatant and the corona-dissociated pellet fraction. IL-6 was depleted by 33.2% in the supernatant with 15.8% recovered in the pellet lysate. TNF-a was depleted by 18.79% in the supernatant with 7.8% recovered in the pellet lysate. (C) Recovery efficiency calculated as the percentage of depleted cytokines traced to the corona pellet lysate (recovered/depleted x100) for IL-6 (15.8%/33.2% x100 = 47.6%) and TNF-α (7.8%/18.79% x100 = 41.5%). This corresponding depletion and recovery confirms that both cytokines are physically captured on the surface of TCNP *via* corona formation.

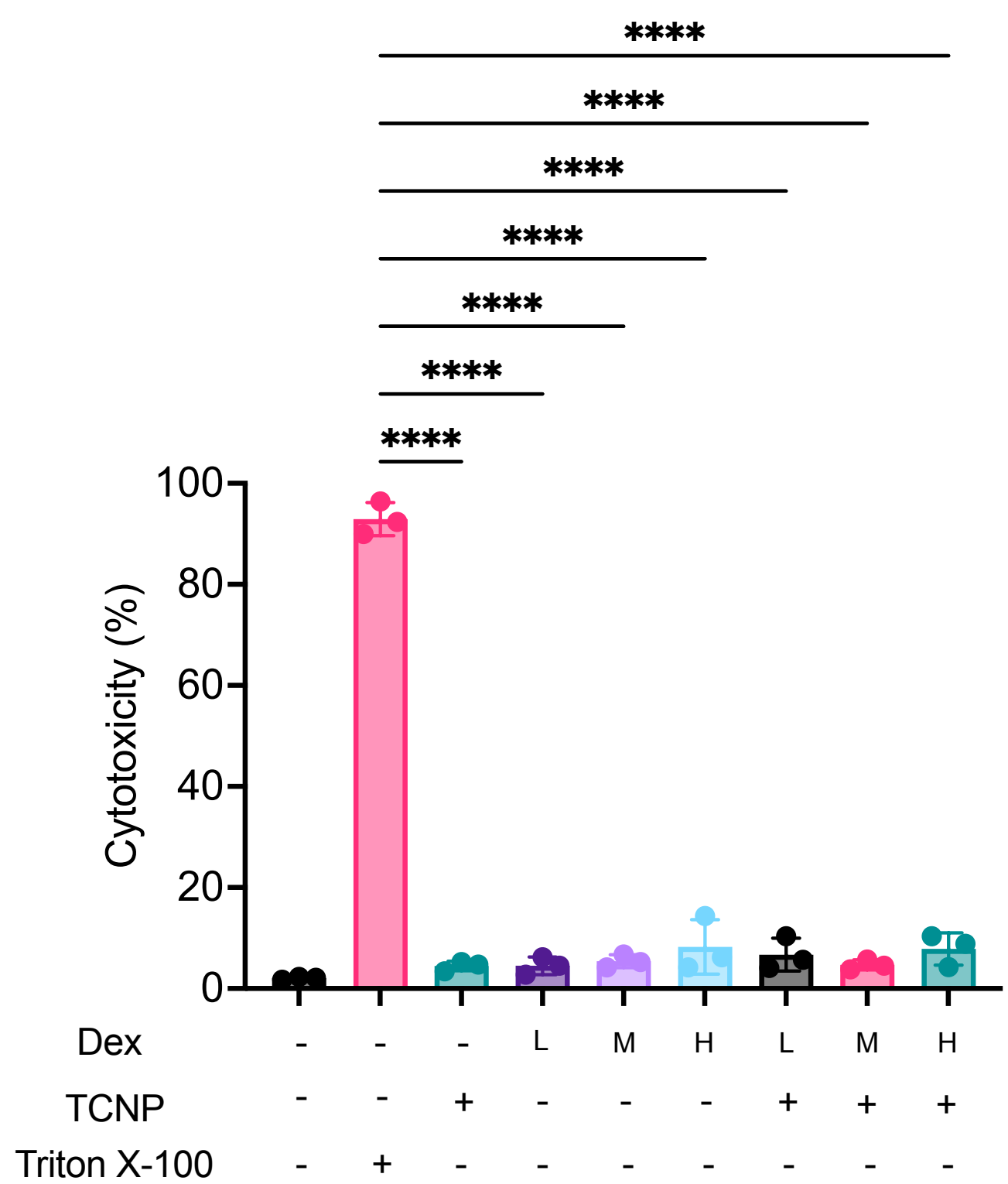

Figure S4. Cytotoxicity was assessed by lactate dehydrogenase (LDH) release assay. Cells treated with three concentrations (Low, Medium, High), either alone or in combination with Dex, showed no significant effect compared to untreated control, indicating TCNP formulations are non-toxic under the tested conditions. Triton X-100 was used as a positive control. Data are expressed as mean ± SD (n=3). ****P < 0.0001 versus Triton X-100 control group.

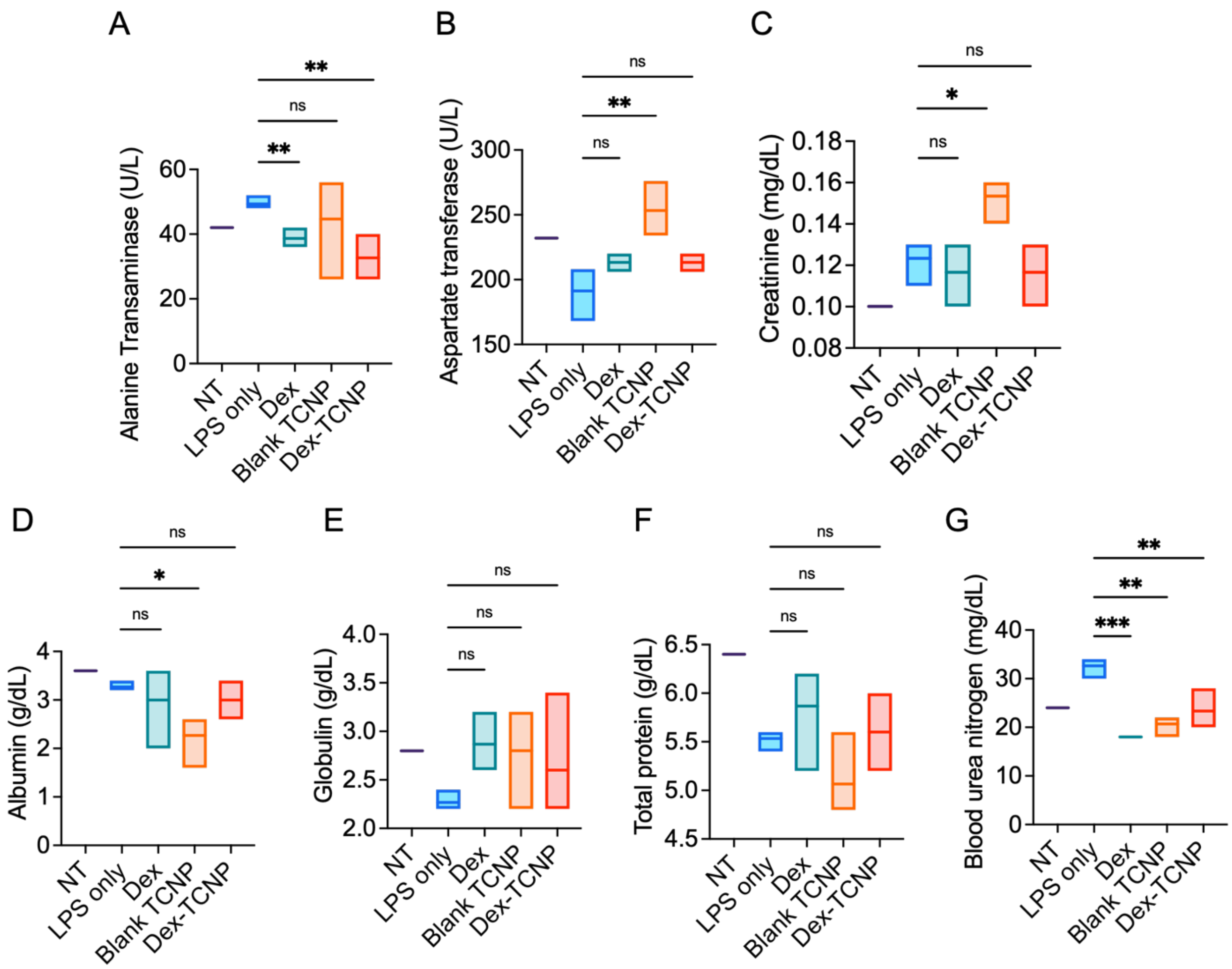

Figure S5. Blood biochemistry was assessed to evaluate hepatotoxicity, nephrotoxicity and overall organ function recovery following treatment. The analysis displays Dex-TCNP treatment reduced the (A) ALT and (G) BUN levels, while no substantial changes were observed in (B) Aspartate Transferase, (C) Creatinine, (D) Albumin, (E) Globulin, (F) Total protein, indicating preserved organ function and tissue recovery. Data are expressed as mean ± SD (n=3). *p < 0.05, **p < 0.01, ***p < 0.001 versus LPS control group.
